# DNA-damage-associated protein co-expression network in cardiomyocytes informs on tolerance to genetic variation and disease

**DOI:** 10.1016/j.isci.2025.112474

**Published:** 2025-04-18

**Authors:** Omar Darrel Johnson, Sayan Paul, José Angel Gutiérrez, William Kent Russell, Michelle Claire Ward

**Affiliations:** 1 Biochemistry, Cellular and Molecular Biology Graduate Program, University of Texas Medical Branch, Galveston, TX 77555, USA; 2 MD-PhD Combined Degree Program, University of Texas Medical Branch, Galveston, TX 77555, USA; 3 Department of Biochemistry and Molecular Biology, University of Texas Medical Branch, Galveston, TX 77555, USA

**Keywords:** Molecular network, Integrative aspects of cell biology, Proteomics

## Abstract

Cardiovascular disease (CVD) is associated with genetic variants and environmental factors. A consequence of multiple risk factors is DNA damage. To examine how DNA damage influences the cardiomyocyte proteome and its relationship to CVD risk, we treated human induced pluripotent stem cell (iPSC)-derived cardiomyocytes with the DNA-damaging agent doxorubicin (DOX). A network constructed from 4,178 proteins reveals 12 co-expressed modules with 403 hub proteins. Five modules correlate with DOX and associate with RNA processing, chromatin regulation, and metabolism. DOX-correlated hub proteins are depleted for proteins that vary in expression across individuals due to genetic variation but are enriched for proteins encoded by loss-of-function intolerant genes. While not enriched for known CVD risk proteins, DOX-correlated hub proteins are enriched for the physical protein interactors of CVD risk proteins. These data demonstrate that protein connectivity in DNA-damage-associated modules influences the tolerance to genetic variation and supports the use of dynamic networks to explore complex traits.

## Highlights

• DNA damage affects protein co-expression in cardiomyocytes
• Damage-responsive hub proteins are depleted for protein quantitative trait loci
• Damage-responsive hubs are enriched for mutation-intolerant proteins
• Damage-responsive hubs are enriched for heart disease risk protein interactors

## Graphical abstract

**Figure:**
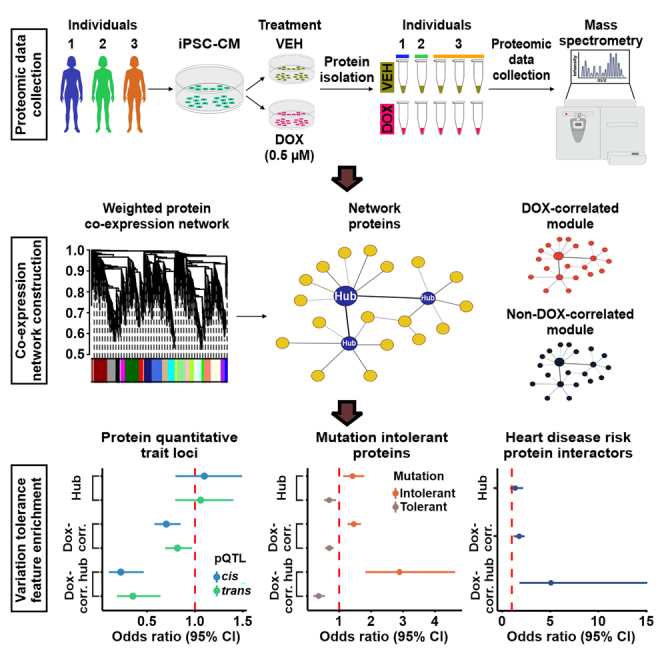

## Introduction

Cardiovascular disease (CVD) is the leading cause of mortality globally.^1^ CVD risk factors including age, diagnosis of metabolic disease, and treatment with chemotherapeutics can result in oxidative stress and apoptosis, leading to accumulated DNA damage and activation of the DNA damage response in the heart.^2^ The amount of DNA damage in the myocardium, as determined by the DNA double-strand break (DSB) marker γH2AX, is predictive of heart failure.^3^ Persistent DNA damage in cardiac cell types can lead to disease and has been detected among some of the most prevalent CVDs, including heart failure and atrial fibrillation.^4^ The myocardium is composed of cardiomyocytes that produce the contractile force of the heart necessary to circulate oxygenated blood throughout the body, and therefore damage to these cells can lead to cardiac dysfunction. Adult human cardiomyocytes are also particularly susceptible to DNA damage, given that they are post-mitotic and unable to regenerate.^4^ This means that DNA damage, induced through DSBs, can only be repaired through error-prone non-homologous end joining, unlike proliferative cell types that can also repair DNA through homologous recombination.^5^

Doxorubicin (DOX) is an effective anthracycline chemotherapeutic that can adversely induce cardiac dysfunction through the formation of DSBs in cardiomyocytes.^6^ This is primarily mediated through its interaction with the DNA topology regulator topoisomerase II (TOP2). Physiologically, the TOP2A and TOP2B isoforms resolve torsional stress in DNA by DSBs; however, in the presence of DOX, TOP2 is trapped on DNA where it generates DNA lesions.^7^ The predominant TOP2 isoform expressed in the heart is TOP2B. TOP2B has been shown to mediate the cardiotoxic effects of DOX in *in vivo* animal models and *in vitro* human disease models.^8^^,^^9^ While DOX can lead to cellular effects through mechanisms including the generation of ROS, at clinically tolerated sub-micromolar doses, DSBs induced through interactions with TOP2B are the main contributors to DOX-induced cardiotoxicity.^10^ Clinically, DOX-induced cardiac dysfunction is not a unique pathology but shares characteristics of multiple CVDs.^6^ For example, 9% of individuals receiving DOX exhibit reductions in their left ventricular ejection fractions within values that would constitute heart failure.^11^^,^^12^ Similarly, treatment with DOX increases the risk for electrophysiologic dysfunction and atrial fibrillation by 10-fold and is associated with other related clinically measurable phenotypes, such as an increased QT-interval.^13^^,^^14^ These pathologies overlap with those influenced by DNA damage and are impacted by genetic risk.^4^^,^^15^

Genome-wide association studies (GWASs) have identified hundreds of risk loci associated with complex CVDs including atrial fibrillation, heart failure, and clinical cardiovascular phenotypes, highlighting the genetic component of CVD.^15^ Although GWASs identify genetic risk loci that can be mapped to genes that implicate putative regulatory effects, they do not explain the molecular mechanisms of the diseases that they associate with.^16^^,^^17^ This ultimately impedes our understanding of the effect of genetic variation on CVD and its applicability to identify potential drug targets.^17^

One approach to understand a complex disease phenotype is to construct networks based on molecular phenotypes such as global mRNA or protein expression levels. For example, while transcriptome profiling of human brain regions has provided insight into cell-type specificity for the risk for neuropsychiatric diseases,^18^ it has been shown that expression networks differ at the mRNA and protein level and that many complex disease phenotypes are observed only at the proteome level.^19^^,^^20^^,^^21^ Targeted studies investigating the protein interactomes of proteins encoded in GWAS loci have identified convergent points in the interaction network for autism spectrum disorder and coronary artery disease, highlighting disease-relevant biology.^22^^,^^23^ Complex disease networks are likely to exert tissue- and cell-type-specific effects. Indeed, proteins relevant to late onset Alzheimer disease are localized within glial cells in the brain.^21^ Similarly, the appropriate cellular context, such as cell type and state, is important for understanding the basis of CVD. Clinically observed CVDs such as DOX-induced cardiotoxicity can be recapitulated in human induced pluripotent stem cell (iPSC)-derived cardiomyocyte models,^24^ allowing for the study of disease-relevant states such as exposure to DOX and hypoxia to understand CVD risk.^25^^,^^26^^,^^27^ However, a protein network has not been generated in this context to understand the interplay between a cellular stressor relevant to CVD and proteins encoded by genes that are implicated in complex CVDs.

We therefore designed a study to determine the effects of the DNA-damaging drug, DOX, on the proteome of human cardiomyocytes. We differentiated cardiomyocytes from iPSCs from three healthy individuals, treated them with a sub-lethal dose of DOX, and measured global protein expression levels. We construct a protein expression network consisting of co-expression modules correlated with DNA-damaging treatment, define the tolerance of network components to genetic variation, and localize CVD risk proteins and their interactors within the network.

## Results

### iPSC-CM proteome resembles the heart ventricle proteome and is affected by DNA damage

**Figure 1:**
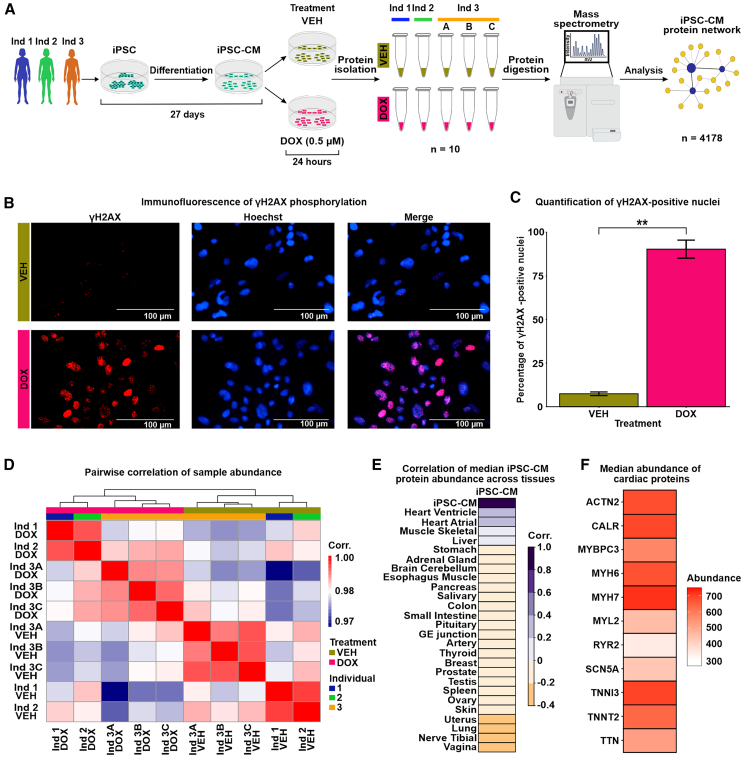
iPSC-CM protein samples cluster by DOX treatment and most closely resemble the heart ventricle proteome (A) Flowchart representing the study design. iPSCs from three individuals (Ind), Ind 1 (blue), Ind 2 (green), and Ind 3 (orange), were differentiated into cardiomyocytes (iPSC-CMs). iPSC-CMs were exposed to 0.5 μM doxorubicin (DOX) or a vehicle control (VEH) for 24 h. The treatment was replicated in Ind 3 three times, yielding 10 total samples. Peptides were isolated and quantified by mass spectrometry, allowing the construction of an iPSC-CM network from 4,178 proteins. (B) Immunostaining of the DNA damage marker, γH2AX, and Hoechst nuclear stain in VEH- and DOX-treated iPSC-CMs. Scale bar: 100 μm (C) Percentage of VEH- and DOX-treated iPSC-CMs that stain positive for γH2AX. Data representative of treatment experiments from three individuals. Data are presented as mean ± SD. Asterisk represents a statistically significant change in γH2AX expression (∗∗*p* < 0.01). (D) Pairwise Pearson correlation of the median protein abundance across all 10 samples. (E) Pearson correlation of the median iPSC-CM protein abundance for all proteins across all experimental samples to the median abundance of those proteins across different human postmortem tissues.^20^ (F) Median protein abundance across experimental samples of select proteins known to be elevated in heart tissue in comparison to other tissue types.

We differentiated iPSCs from three healthy female donors into cardiomyocytes (iPSC-CMs) using biphasic WNT modulation (Figure 1A and Table S1). iPSC-CMs were metabolically selected and matured for 27 days post-differentiation initiation (See STAR Methods).^26^ Flow cytometry analysis of two individuals indicated high-purity cultures with a median of 97% of cells expressing cardiac troponin T (Figure S1).

To determine the effect of DNA damage on the cardiomyocyte proteome, we treated iPSC-CMs with a TOP2B-inhibiting, clinically relevant concentration of DOX (0.5 μM) and a water vehicle control (VEH) for 24 h. This dose of DOX causes minimal cell death in iPSC-CMs but induces thousands of mRNA expression changes for genes in pathways related to p53 signaling, base excision repair, and DNA replication.^28^ We confirmed the DNA-damaging effects of DOX under these conditions by assaying the expression of the DNA DSB marker γH2AX (Figure 1B). DOX-treated cardiomyocytes have significantly higher γH2AX expression compared to VEH-treated cardiomyocytes (Figure 1C; 90% vs. 7%; t test; *p* < 0.05). To account for technical variability in the drug treatment and proteomic data collection, the DOX and VEH treatment in iPSC-CMs from one individual was replicated three times, resulting in a total of 10 samples across individuals and treatments.

Global protein expression data were inferred from peptide identification and quantification using data-independent acquisition mass spectrometry (DIA; see STAR Methods). Peptides were mapped to 4,261 proteins present in at least one sample (Data S1). Four non-human proteins and proteins that were present in less than half of the samples were filtered out. To enable construction of as complete a network as possible, we used the remaining 4,178 proteins to impute protein abundance data for the 246 proteins with missing data using a feature-clustering-based imputation method commonly used for proteomics data (Figure S2A).^29^^,^^30^ On average, imputed proteins are less abundant than proteins present in all 10 samples (median log_2_ abundance all = 19.83, median log_2_ imputed abundance = 16.98; Figure S2B). We took advantage of the technical replicates to remove unwanted variation in the data (see STAR Methods).^31^ After correction, principal-component analysis reveals that PC1, which accounts for 32% of variation in the data, associates with drug treatment, whereas PC2, accounting for 29% of the variation in the data, associates with individual (Figures S2C and S2D). Similarly, when comparing all pairwise sample correlations, the data primarily separate into two clusters corresponding to DOX and VEH treatment (Figure 1D).

To gain insight into the utility of our iPSC-CM proteome data for understanding the effect of DNA damage on the heart, we correlated the median expression of our set of proteins with the expression of proteins measured in 26 postmortem human tissues from hundreds of individuals.^20^ The iPSC-CM proteome is most similar to the proteome from heart left ventricle and atrial appendage, followed by skeletal muscle (Figure 1E). iPSC-CMs express cardiac-specific and cardiac-abundant proteins, including myosin heavy chain 7 (MYH7), myosin heavy chain 6 (MYH6), and troponin I (TNNI3; Figure 1F). Together, these findings affirm that the proteome of our iPSC-CMs closely resembles ventricular and atrial tissue, includes key cardiomyocyte proteins, and demonstrates the influence of DNA damage as the main contributor to variation of protein abundance values within our experimental model.

### Network analysis reveals DOX-correlated modules, response proteins, and hub proteins

**Figure 2:**
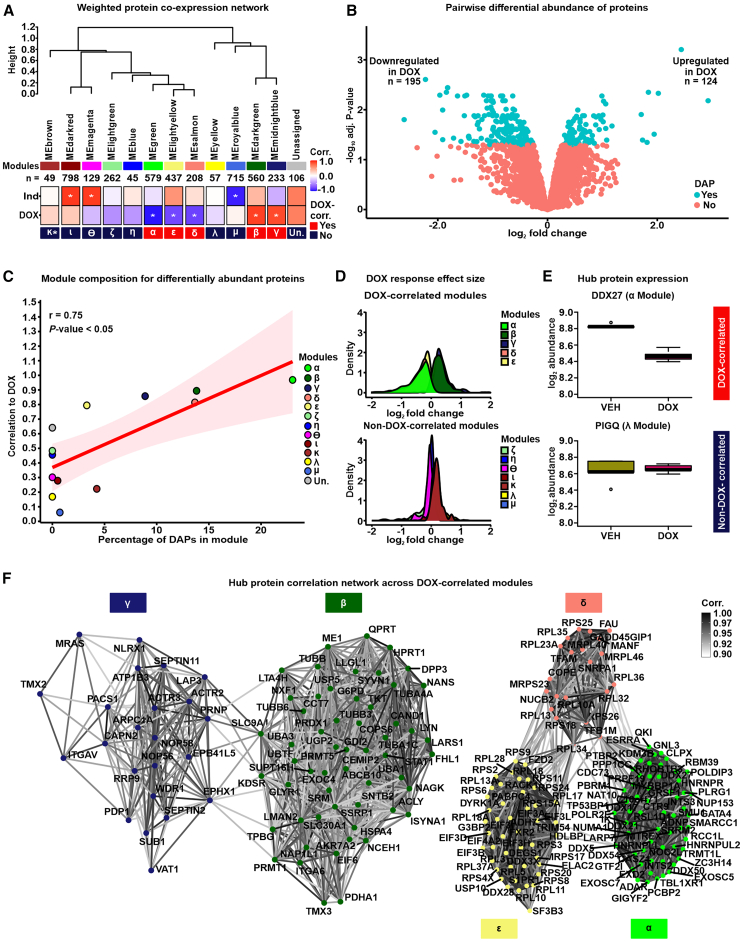
Network analysis of the iPSC-CM proteome identifies protein co-expression modules correlated with DOX treatment (A) Hierarchical clustering of 12 co-expression module eigen (ME) proteins based on their Pearson correlation. Height represents the dissimilarity between ME proteins (1-corr.). Each module is represented by a color and the number of proteins in the module specified. The "Unassigned" module, shaded gray, includes proteins that cannot be represented by one of the 12 ME proteins. The correlation between each ME protein and the known biological variables: Individual (Ind) and DOX treatment is shown. Asterisk represents a significant correlation between the ME protein and the trait (∗*p* < 0.01). Modules are designated by Greek letters in order of decreasing correlation with DOX and summarized as DOX-correlated modules (red) and non-DOX-correlated modules (dark blue). (B) Volcano plot representing proteins that are differentially abundant (DAPs; adjusted *p* < 0.05; blue) and not differentially abundant (salmon) between VEH- and DOX-treated iPSC-CMs. (C) Correlation between the proportion of DAPs in a module and the correlation of the module to DOX. The best-fit line together with 95% confidence interval is shown. (D) Distribution of effect sizes of response to DOX treatment (log_2_ fold change from pairwise differential abundance model) for the five DOX-correlated and seven non-DOX-correlated modules. (E) Examples of hub protein abundance values in VEH- and DOX-treated iPSC-CMs in a DOX-correlated module (α; DDX27) and a non-DOX-correlated module (λ; PIGQ). (F) DOX-correlated hub protein co-expression correlation network, where nodes are hub proteins and edges represent the weighted correlation between them. Connections among DOX-correlated proteins with a correlation of ≥0.9 are depicted for visualization.

In order to identify sets of co-expressed proteins within our data, we utilized weighted correlation network analysis (WGCNA).^32^ WGCNA assumes the co-expression network follows a scale-free topology, where few nodes (proteins) have high connectivity and many nodes have low connectivity, and requires a soft power threshold to determine the weights of edges connecting nodes. We regressed total network connectivity on the connectivity frequency distribution at different power thresholds and determined that the lowest power threshold that yielded the best fit to a scale-free network (0.71 regression coefficient) was 20 (Figure S3A). The scale-free fit is further supported by decreasing connectivities at higher power thresholds, where we find that at a power threshold of 20, our network has a median connectivity score of 53.6, mean connectivity of 65.4, and maximum connectivity of 216 (Figure S3B). Using these criteria, we generated a network comprising 21 co-expressed modules with at least 40 proteins per module (Figure S3C), summarized by 21 eigen proteins (Figure S3D). All combinations of proteins in the co-expression network are summarized as pairwise correlations (Data S2). To better distinguish individual modules, we merged modules with highly correlated eigen proteins (Pearson correlation >0.85) and overlapping gene ontologies, which collapsed the data into 12 distinct modules (Figures 2A and S3E–S3G). These modules contain between 45 and 798 proteins (Table S2) and represent diverse gene ontologies (Table S3). One hundred six proteins (2.5% of all expressed proteins) do not fit the profile of any module and were categorized as “unassigned” (Table S2). These proteins are not co-expressed and have lower intra-modular connectivity (kIN) than the median kIN across assigned modules (normalized kIN unassigned = 0.015, normalized kIN assigned = 0.06; Wilcoxon rank-sum test; *p* < 0.05). This set of proteins is not enriched for any gene ontology. Imputed proteins are distributed across modules representing 1.6%–16% of all proteins within a module. We associated each module with the two biological features of our data, namely DOX treatment and Individual (IND). To do so, we measured the correlation between each module’s eigen protein and either IND or DOX treatment (Figure 2A). We named each of the 12 modules based on the order of their absolute correlation with DOX treatment: α, β, γ, δ, ε, ζ, η, θ, ι, κ, λ, and μ. Eigen proteins from the α, β, γ, ε, and δ modules exhibited significant correlations with DOX treatment (Pearson correlation; *p* < 0.01) and were therefore categorized as DOX-correlated modules. Modules α, δ, and ε have strong negative DOX correlations of −0.97, −0.81, and −0.79, whereas modules β and γ have similar positive correlations of 0.89 and 0.86, respectively. In contrast, eigen proteins from the μ, ι, and θ modules are significantly correlated with IND. This delineation indicates that 5 of the 12 co-expressed modules in our iPSC-CM protein abundance correlation network are specifically associated with DOX treatment.

To support the categorization of five modules as DOX-correlated, we performed an independent pairwise differential abundance (DA) test. Using this approach, we identified 319 DA proteins among the 4,178 evaluated proteins (adjusted *p* < 0.05; Table S2; see STAR Methods). One hundred twenty-four DA proteins exhibit increased abundance in the DOX-treated iPSC-CMs, whereas 195 DA proteins show decreased abundance (Figure 2B). The percentage of DA proteins in a module correlates with the module’s correlation to DOX (rho = 0.75; *p* < 0.05; Figure 2C). Similarly, proteins within DOX-correlated modules tend to have a greater response to DOX treatment as measured by their log fold change, compared to proteins in non-DOX-correlated modules (Figure 2D).

In order to test the robustness of our approach for identifying DOX-responsive proteins, we also acquired protein measurements of the same samples by data-dependent acquisition (DDA) on the mass spectrometer (see Methods). We identified 4,501 proteins using this method. All proteins identified by DIA were also identified by DDA, and the abundance of the 3,027 proteins present across all samples in both datasets is highly correlated (rho = 0.75; *p* < 0.001; Figure S4). Following imputation and unwanted variance removal of the DDA data, we identified 223 DA proteins among 3,954 proteins. The effect size of the response to DOX treatment among proteins included in both acquisition methods is correlated (Pearson correlation coefficient = 0.35; *p* < 0.001; Figure S5), and the proportion of DA proteins is similar (7% for DDA and 8% for DIA). These results suggest that protein abundance changes in response to DOX identified by DIA are replicated when abundance data are collected through data-dependent acquisition.

We identified a set of proteins with a high level of intra-modular connectivity that are likely to play a central role in the biological processes associated with each module. These 403 hub proteins have the highest correlation with the module eigen proteins and the highest intra-modular connectivity (kIN) score (top 10% of all network proteins; see STAR Methods; Figure S6 and Table S2). Hub proteins predominantly reflect the module’s collective response to DOX or VEH treatment. For example, DDX27 in the α module is downregulated in response to DOX treatment, whereas PIGQ in the λ module shows no difference in abundance in response to DOX (Figure 2E). We then focused on the relationship between hub proteins across the five DOX-correlated modules (*n* = 202). The resulting network revealed not only strong intra-modular connections but also significant inter-modular correlations among proteins with a similar direction of effect in response to DOX treatment, indicating potential roles in inter-modular overlap for biological processes (Figure 2F).

### Module proteins differ in their tissue specificity and cellular localization

Having identified modules of co-expressed proteins that are correlated with DOX treatment, we next sought to investigate the properties associated with each module. To determine the specificity of module proteins to heart tissue, we utilized data from the Human Protein Atlas (HPA) and the Genotype-Tissue Expression (GTEx) projects.^20^^,^^33^ These resources provide extensive gene and protein expression measurements across various tissues. The HPA database highlights 419 genes with elevated expression in heart tissue, defined as at least a 4-fold higher mRNA level in the heart compared to the average in other tissues. We detect 277 proteins corresponding to heart-elevated genes in our data. Heart-elevated proteins constitute only a small proportion of proteins in each module regardless of DOX-correlation status (median all = 4.5%; range all = 0%–8%; Figure S7A). For a quantitative analysis of tissue specificity of DOX-correlated module proteins, we obtained tissue specificity (TS) scores for proteins in heart left ventricle tissue from GTEx. For each module in the network, module TS scores were compared to the mutually exclusive set of all other network proteins (network TS median = 0.33). We find significant deviations in TS scores from DOX-correlated modules α, β, γ, and δ, as well as non-DOX-correlated modules κ and ι (Wilcoxon rank-sum test; *p* < 0.05). Modules β, δ, κ, and ι have higher TS scores, whereas α and γ have lower TS scores (Figure S7B). While the module with the strongest correlation to DOX, α, contains proteins that are the least specific to heart ventricle tissue (TS score = −0.47), there is no correlation between tissue specificity and correlation to DOX (rho = 0.18; Figure S7C). Both DOX-correlated proteins and DOX-correlated hub proteins are neither enriched nor depleted in the set of 419 elevated heart proteins compared to non-DOX-correlated proteins and hub proteins (Figure S7D). These findings suggest that while there may be heterogeneous tissue-specific effects of proteins at the modular level, proteins that are central to the DNA damage response in cardiomyocytes are not specific to the heart ventricle.

We next asked whether proteins within each module are restricted to specific intracellular or extracellular locations. We first investigated the localization of module proteins across four broad categories: intracellular, membrane-bound, plasma-detected, and secreted proteins as defined by HPA. We find that DOX-correlated modules α, β, and δ contain proteins that are enriched in the intracellular category compared to proteins not contained within each of these modules (Fisher’s exact test; odds ratio [OR] = 1.8, 1.3, and 1.5, respectively; *p* < 0.05; Figure S8A). Module γ is the only DOX-correlated module enriched for membrane proteins (OR = 1.4; *p* < 0.05), along with non-DOX-correlated modules η, λ, and μ. Plasma-detected proteins are only enriched in module β (OR = 1.61; *p* < 0.05), whereas secreted proteins are only enriched in module μ (OR = 2.3; *p* < 0.05). The ε module is the only DOX-correlated module not enriched for any category. These results suggest that the cellular localization of proteins differs across modules. We then asked whether the sub-cellular localization of intracellular proteins differs across modules using annotation data from the UniProt database.^34^ Network proteins are generally distributed across multiple sub-cellular organelles including the sarcomere, nucleus, mitochondrion, lysosome, Golgi apparatus, endoplasmic reticulum, cytoskeleton, cytoplasm, cell membrane, and autophagosome (Figure S8B). Nuclear proteins are enriched in modules α, β, and ε (Fisher’s exact test; *p* < 0.05). Modules α and ε also share enrichment for cell membrane, cytoplasm, endoplasmic reticulum, and mitochondrial and lysosomal proteins. Module δ is enriched for mitochondrial proteins, whereas γ is enriched for endoplasmic reticulum and cell membrane proteins.

### DOX-correlated module proteins are enriched for distinct biological processes

To elucidate the broad functional roles of proteins within the DOX-correlated modules, we tested whether proteins associated with distinct biological processes are enriched in each module (see STAR Methods). We found that the α, β, δ, and ε modules show enrichment for various biological processes (Fisher’s exact test; adjusted *p* < 0.05; Figures S9 and S10). The α module is enriched for 160 unique processes related to the DNA damage response, gene regulation via RNA splicing and metabolism, as well as chromatin regulating processes such as histone methylation and acetylation (Table S3). The β module is uniquely enriched for 35 processes related to protein localization within the nucleus and metabolism of carbohydrates, organophosphates, and oxoacids. Proteins in the δ module are uniquely enriched for 13 processes related to mitochondrial gene expression, translation, and ATP synthesis. The ε module is uniquely enriched for 26 processes related to DNA replication and chromatin assembly. There are no processes significantly enriched within the γ module; however, the most represented processes include glutamine family amino acid catabolic processes and ion homeostasis. Biological processes shared across modules include cytoplasmic translation (enriched in δ and ε), post-transcriptional regulation of gene expression (α and ε), and ribosome and ribonucleoprotein complex biosynthesis (α, ε, and δ). Biological process enrichment testing using only hub proteins of each module identified similar enrichment patterns to those in the complete module (for example, RNA metabolism and processing in the α module), indicating that the set of module hub proteins captures the core set of biological processes most representative of the entire module. Together, these data show a diversity of processes enriched among DOX-correlated modules.

We then asked whether proteins in each module are enriched for specific protein families consistent with their distinct biological processes. The α module is enriched for the splicing factor SR family and Rnase PH family (Fisher’s exact test; adjusted *p* < 0.05; Figure S11), whereas β is enriched for the TCP-1 chaperonin family, and δ is enriched for signaling proteins in the 14-3-3 family. Domain-enriched modules therefore generally corroborate the biological process enrichment analysis results.

We selected five key protein categories to investigate further based on our module-specific functional enrichment results (Figure S12A; see STAR Methods).^20^^,^^35^^,^^36^^,^^37^ The α module is uniquely enriched for transcription factors (Fisher’s exact test; OR = 5.6; adjusted *p* < 0.05; Figure S12B), particularly the homeodomain, GATA, and C2H2 Zinc Finger families that include the GATA4, MEIS1, and ZNF629 transcription factors (Figure S12C). Mutations in GATA4 are associated with atrial septal defects, arrhythmia, and a reduced capacity for the cardiac hypertrophic response^38^ and have been implicated in DOX-induced cardiotoxicity.^39^ Both the α and ε modules show enrichment for stress granule components and RNA-binding proteins (adjusted *p* < 0.05; Figure S12A). RNA-binding proteins related to multiple post-transcriptional processes including splicing, translation, and mRNA stability are enriched (Figure S12D) and include proteins such as QKI, YBX3, and GNL3 (Figure S12E). QKI regulates pre-mRNA splicing, export of mRNAs from the nucleus, protein translation, and mRNA stability^40^ and is implicated in cardiomyocyte calcium dynamics and contractility,^41^ cardiomyopathies,^40^ and attenuation of DOX-induced cardiotoxicity.^42^ The β module uniquely exhibits enrichment for enzymes (OR = 1.9; adjusted *p* < 0.05; Figure S12A) including glutathione metabolism, fatty acid activation, and drug metabolism (Figure S12F). The most DOX-responsive enzymes include NDUFB7, a member of the δ module and critical component of the mitochondrial membrane respiratory chain complex I (Figure S12G). These results are consistent with the biological processes enriched in each module.

### Hub proteins in DOX-correlated modules are depleted for pQTLs

**Figure 3:**
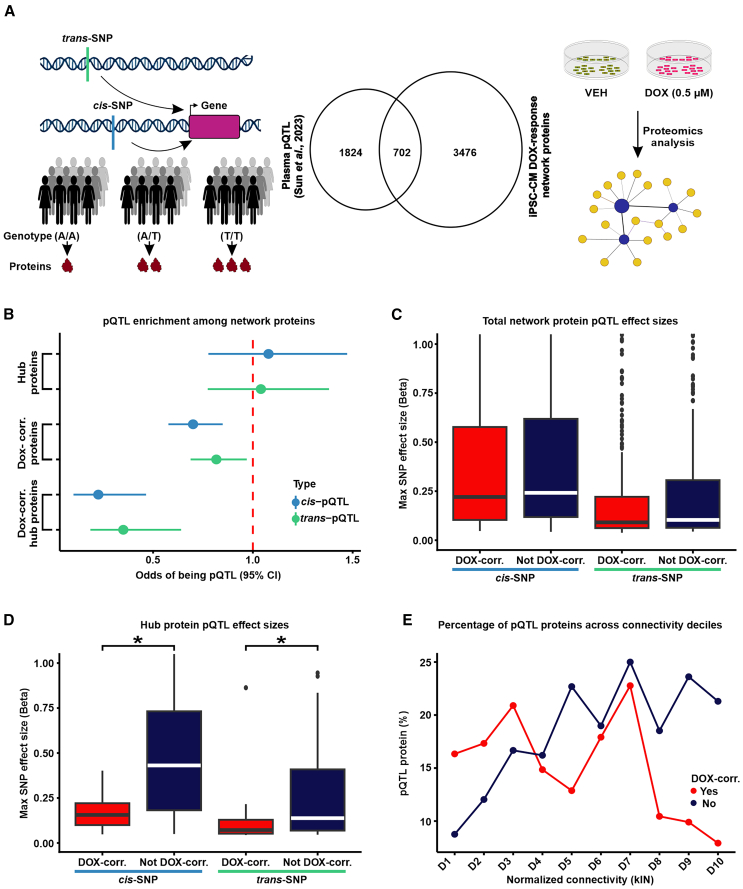
DOX-correlated hub proteins are depleted for protein quantitative trait loci (A) Schematic representing the rationale behind the test design. Protein expression can be influenced by *cis*- or *trans*-SNPs, resulting in differential abundance of proteins across individuals with different genotypes. We integrated existing protein quantitative trait loci (pQTL) data from plasma samples^17^ with our DOX-treated iPSC-CM protein network. (B) Enrichment of *cis*- (blue) and *trans*-pQTLs (green) among hub proteins, DOX-correlated proteins, and DOX-correlated hub proteins. (C) Maximum pQTL effect sizes for *cis*- and *trans*-pQTLs among all proteins in DOX-correlated modules and non-DOX-correlated modules. (D) Maximum pQTL effect sizes for *cis*- and *trans*-pQTLs among all hub proteins in DOX-correlated modules and non-DOX-correlated modules. Asterisk represents a significant difference in effect sizes between DOX-correlated and non-DOX-correlated modules (∗*p* < 0.05). (E) Percentage of pQTL proteins across connectivity deciles for normalized kIN for proteins in DOX-correlated (red) and non-DOX-correlated (blue) modules. Deciles are ordered by increasing kIN scores.

We next focused our attention on the properties of the 403 hub proteins within the network. We were particularly interested in understanding the tolerance of these central proteins to physiological genetic variation. To do so, we investigated proteins whose expression level varies across individuals depending on the genotype of an associated SNP, i.e., protein quantitative trait loci (pQTLs; Figure 3A). Prior research focused on serum proteins has shown that highly connected proteins central to co-expression networks are typically depleted for pQTLs.^43^ However, how network centrality interacts with the dynamic molecular response to perturbation to shape the distribution of pQTLs is not known. We therefore obtained a set of pQTLs identified in human plasma from thousands of individuals^17^ and overlapped these with iPSC-CM network proteins (Table S2). We first asked whether pQTL proteins associated with either *cis-* or *trans*-SNPs are enriched among hub proteins. Hub proteins are neither enriched nor depleted for pQTLs (Fisher’s exact test; OR = 1.1; 95% confidence interval [CI] = 0.8–1.5 for *cis*-pQTLs and OR = 1.0; 95% CI = 0.8–1.4 for *trans*-pQTLs; Figure 3B). We then asked whether pQTL proteins are enriched among proteins that respond to DNA damage. DOX-correlated proteins are modestly depleted for pQTL proteins associated with either *cis-* (OR = 0.7; 95% CI = 0.6–0.8; *p* < 0.05; Figure 3B) or *trans*-SNPs (OR = 0.8; 95% CI = 0.7–1.0; *p* < 0.05, Figure 3B). However, DOX-correlated hub proteins show an even more pronounced depletion for pQTL proteins mapped to *cis-* (OR = 0.2; 95% CI = 0.1–0.5; *p* < 0.05; Figure 3B) or *trans*-SNPs (OR = 0.4; 95% CI = 0.2–0.6; *p* < 0.05; Figure 3B).

Next, we investigated those pQTLs that correspond to proteins expressed within our network and asked whether the SNP effect size of the pQTLs differed across network components. We assigned each pQTL protein to the *cis-* or *trans*-SNP with the greatest effect size. As expected, the median effect size for *cis*-pQTL proteins in the network is greater than that for *trans*-pQTLs (0.2 vs. 0.1). There is no difference in the *cis*- or *trans*-pQTL effect sizes between DOX-correlated and non-DOX-correlated proteins (Figure 3C). However, DOX-correlated hub proteins have lower *cis-* and *trans*-pQTL effect sizes than hub proteins that are not correlated with DOX (Wilcoxon rank-sum test; *p* < 0.05; Figure 3D).

Given the depletion of pQTLs among DOX-correlated hub proteins and not the total set of hub proteins, we next asked if there is a relationship between the intra-modular connectivity of a protein and the probability of that protein being a pQTL that is influenced by DOX. We assigned proteins into two groups based on their DOX-correlation status, stratified proteins within each group into deciles based on their connectivity, and calculated the percentage of pQTL proteins in each decile. In the non-DOX-correlated group, the decile with the highest connectivity shows the greatest percentage of pQTL proteins (21%), whereas the decile with the lowest connectivity shows the lowest percentage of pQTL proteins (9%). Across deciles, there is a general upward trend in the percentage of pQTL proteins as intra-modular connectivity increases (Figure 3E). In the DOX-correlated group, the highest connectivity decile shows a reduction in the percentage of pQTL proteins (8%) relative to the lowest connectivity decile (16%) and more variability for the percentage of pQTL proteins across deciles. Therefore, the percentage of pQTL proteins across different deciles showed opposite trends depending on their correlation to DOX. The decrease in pQTL proteins with the highest connectivity among DOX-correlated proteins suggests that hub proteins correlated to DOX treatment are under stronger evolutionary constraints, leading to reduced genetic variation in these highly connected proteins.

We also tested for enrichment of pQTLs across modules. The α module, with the highest correlation to DOX, is depleted for pQTLs (Fisher’s exact test; OR = 0.6; CI = 0.5–0.8; adjusted *p* < 0.05; Figure S13). Conversely, the μ module, with the lowest correlation to DOX, is the only module enriched for pQTLs (OR = 1.4; CI = 1.1–1.7; adjusted *p* < 0.05) and is one of three modules correlated with Individual. There are no modules correlated with both Individual and DOX, which supports differences in tolerance to variation across these protein sets.

In summary, our analysis reveals that DOX-correlated hub proteins are less likely to be associated with pQTLs, indicating their likelihood to play an essential role in maintaining network stability and function under stress conditions.

### DOX-correlated hub proteins are enriched for loss-of-function-intolerant proteins

Given that DOX-correlated hub proteins are depleted for proteins that vary in their expression across healthy individuals, we next asked about the tolerance of these proteins to variation more broadly. We utilized several genetic tolerance scores to assess the functional impact and evolutionary constraint of genes encoding proteins within specific modules. First, we considered the probability of each protein in a module being haploinsufficient (pHaplo), which estimates whether a single copy of a gene can maintain normal function, or triplosensitive (pTriplo), which estimates the risks of gene dose increases, that can be equally detrimental. Genes with a high pHaplo score (≥0.86) or a high pTriplo score (≥0.94) are deemed haploinsufficient or triplosensitive, respectively, and likely precipitate health consequences.^44^ We compared the gene dose scores of DOX-correlated module proteins against the broader network (see STAR Methods). The α and ε modules have higher pHaplo scores compared to network proteins, whereas δ module proteins have lower pHaplo scores (Wilcoxon rank-sum test; *p* < 0.05; Figure S14A). The α module also shows increased pTriplo scores compared to the network average, whereas the δ module’s pTriplo scores are lower (*p* < 0.05; Figure S14B). These results show that the most DOX-correlated module, α, is most sensitive to gene dosage changes.

**Figure 4:**
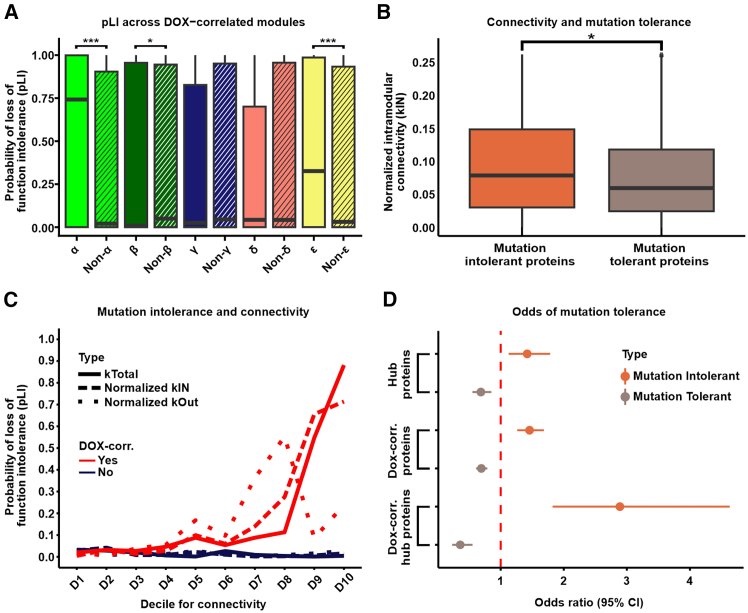
DOX-correlated hub proteins are enriched for loss-of-function-intolerant proteins (A) Probability of loss-of-function intolerance (pLI) for all proteins in each DOX-correlated module (x) and all proteins outside of the module (Non-x). Asterisk represents a significant difference between x and Non-x (∗*p* < 0.05; ∗∗∗*p* < 0.001). (B) Connectivity (normalized kIN) among mutation-tolerant (pLI ≤0.1) and mutation-intolerant (pLI ≥0.9) proteins. Asterisk represents a significant difference in scores between module-specific proteins and all proteins outside of modules (∗*p* < 0.05). (C) Median pLI scores across proteins in connectivity deciles (kTotal (solid line), normalized kIN (dashed line), and normalized kOut (dotted line)) for proteins in DOX-correlated modules (red) and non-DOX-correlated modules (blue). Deciles are ordered by increasing connectivity scores. (D) Enrichment of mutation-intolerant (orange) and mutation-tolerant (brown) proteins among hub proteins, DOX-correlated proteins, and DOX-correlated hub proteins.

Next, we examined the tolerance of module proteins to mutations that reduce or eliminate protein function. Genes with a high probability (≥0.9) of loss-of-function intolerance (pLI) are considered loss-of-function intolerant, meaning they cannot withstand loss-of-function mutations without significant phenotypic impact. Conversely, genes with a low probability (≤0.1) can tolerate loss-of-function mutations with minimal phenotypic consequences and are considered loss-of-function tolerant.^44^^,^^45^ Both α and ε module proteins exhibit significantly higher pLI scores than proteins outside these modules (Wilcoxon rank-sum test*; p* < 0.05; Figure 4A and Table S2), indicating that they are less tolerant to genetic variation. In contrast, the β module is characterized by lower pLI scores (*p* < 0.05; Figure 4A), suggesting these proteins can better tolerate loss-of-function mutations. These results emphasize that the α module contains proteins that are highly intolerant to pathogenic variation.

To test the relationship between modular connectivity and pLI across the network, we identified the set of loss-of-function-tolerant and loss-of-function-intolerant proteins. We find that loss-of-function-intolerant proteins have a higher level of intra-modular connectivity (normalized kIN) compared to loss-of-function-tolerant proteins (Wilcoxon rank-sum test; *p* < 0.05; Figure 4B). To understand the relationship between different types of connectivity and DOX-correlation, we next considered the spectrum of tolerance to mutation across all proteins in the network. First, we classified all proteins into two groups based on their DOX-correlation status. We then generated deciles for intra-modular connectivity (kIN), inter-modular connectivity (kOut), and total network connectivity (kTotal) for both DOX-correlated and non-DOX-correlated proteins. For DOX-correlated proteins, there is a marked increase in pLI scores in the highest kTotal and kIN deciles (Figure 4C). Conversely, pLI scores for kOut appeared more variable at higher connectivity deciles. In comparison to DOX-correlated proteins, median pLI scores for non-DOX-correlated proteins remain consistently low across deciles for all measures of connectivity. These findings highlight that DOX-correlated proteins with the highest total connectivity, primarily driven by intra-modular connectivity, are essential for cellular function under DNA damage conditions and are under strong evolutionary constraints to maintain their functional integrity. In contrast, non-DOX-correlated proteins do not exhibit a relationship between connectivity and mutation intolerance, suggesting a less critical role in maintaining network stability.

We next asked whether loss-of-function-intolerant proteins are enriched among proteins central to the protein network given that there are increased pLI scores in the highest decile for intra-modular connectivity. DOX-correlated proteins are modestly enriched for proteins whose encoding genes are loss-of-function intolerant (Fisher’s exact test; OR = 1.5; CI = 1.3–1.7; *p* < 0.05; Figure 4D) and depleted for proteins that are tolerant to mutation (OR = 0.7; CI = 0.6–0.8; *p* < 0.05). Hub proteins are modestly enriched for loss-of-function-intolerant proteins (OR = 1.4; CI = 1.1–1.8; *p* < 0.05) and depleted for mutation-tolerant proteins (OR = 0.7; CI = 0.6–0.9; *p* < 0.05). However, DOX-correlated hub proteins are highly enriched for mutation-intolerant proteins (OR = 2.9; CI = 1.8–4.6; *p* < 0.05) and depleted for mutation-tolerant proteins (OR = 0.4; CI = 0.2–0.6; *p* < 0.05; Figure 4D). Similarly, Individual-correlated module proteins are more tolerant of loss-of-function mutations than DOX-correlated module proteins, and modules correlated with neither Individual nor DOX (Wilcoxon rank-sum test; *p* < 0.05, Figure S14C). These data demonstrate that DOX-correlated hub proteins are likely to be the most critical proteins to the DNA damage response network.

### DOX-correlated modules contain cardiovascular-disease-associated proteins

**Figure 5:**
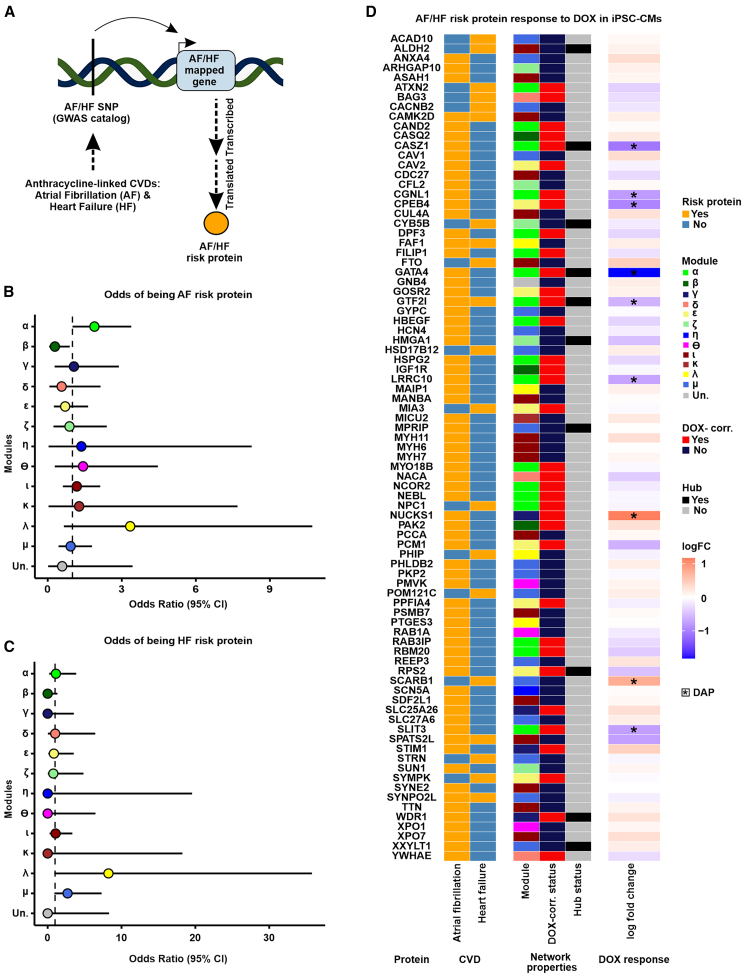
DOX-correlated modules contain proteins associated with risk for atrial fibrillation (A) Schematic representing how risk proteins for the anthracycline-associated CVD traits atrial fibrillation (AF) and heart failure (HF; “heart disease” GWAS catalog ontology class EFO_0003777) are obtained. AF- and HF-associated SNPs (AF/HF-SNP) from GWAS are mapped to nearby genes (AF/HF mapped gene) and converted to the corresponding protein identifier (AF/HF risk protein). (B) Enrichment of 70 expressed atrial fibrillation risk proteins across network modules. (C) Enrichment of 20 expressed heart failure risk proteins across network modules. (D) Response of AF and HF risk proteins to DOX. A positive fold change indicates proteins that increase in expression in response to DOX. Asterisk represents differentially abundant proteins (DAPs; adjusted *p* < 0.05).

DOX treatment in cancer patients is associated with increased risk for atrial fibrillation (AF) and heart failure (HF).^46^^,^^47^^,^^48^^,^^49^ We therefore obtained data for genetic loci associated with these two CVDs from the GWAS catalog^50^ and asked whether these loci are nearexpressed proteins belonging to DOX-correlated modules (Figure 5A). Seventy mapped AF genes and 20 mapped HF genes are expressed as proteins in our iPSC-CM data. The α module shows nominal enrichment for AF proteins (OR = 1.89; CI = 1.0–3.4; nominal *p* < 0.05; Figure 5B) but is not enriched for HF proteins (Figure 5C). No other DOX-correlated module shows evidence for enrichment with either trait. We next investigated the response of individual disease-associated proteins to DOX; 31 of 70 expressed AF risk genes and 6 of 20 expressed HF risk genes are within DOX-correlated modules (Figure 5D). Many AF risk genes in the α module, including CASZ1, CGNL1, GATA4, GTF2I, LRRC10, and SLIT3, are also DAPs that are downregulated in response to DOX. GTF2I is the only DOX-correlated protein that is associated with both AF and HF. NUCKS1 is an AF-associated DAP in the γ module and is therefore upregulated in response to DOX. Transcription factors CASZ1, GATA4, and GTF2I are the only risk proteins for AF or HF in DOX-correlated modules that are both DAPs and hub proteins. These results show that several CVD risk proteins respond to DNA damage.

### DOX-correlated hub proteins are enriched for physical protein-protein interactors of CVD-associated proteins

**Figure 6:**
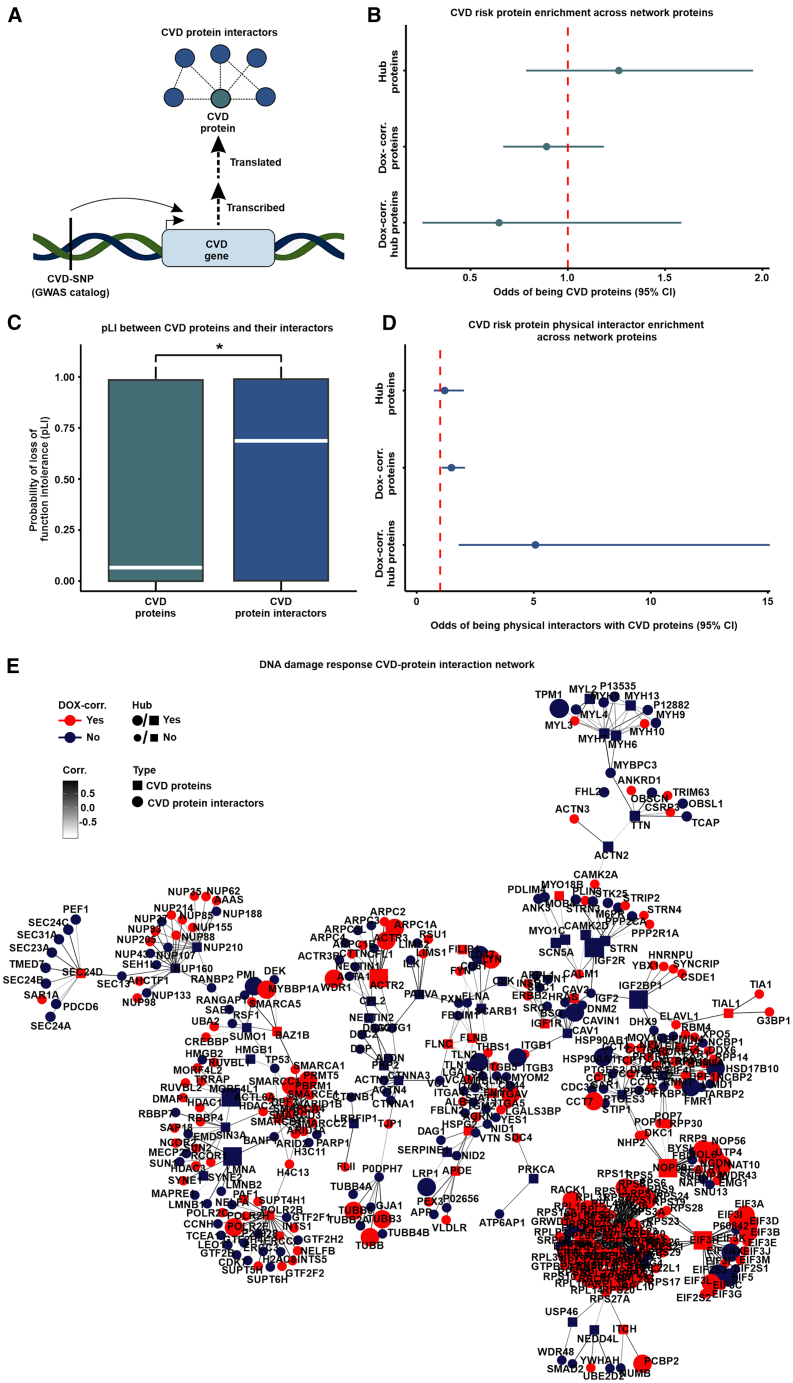
DOX-correlated hub proteins are enriched for physical protein interactors of CVD risk proteins (A) Schematic representing the rationale behind the test design. CVD-associated SNPs (CVD-SNP) are mapped to nearby genes (CVD gene) that are translated into proteins (CVD proteins) that may physically interact with other proteins (CVD protein interactors). (B) Enrichment of CVD risk proteins among hub proteins, DOX-correlated proteins, and DOX-correlated hub proteins. (C) pLI score distribution of CVD risk proteins and CVD risk protein interactors. Asterisk represents a significant difference in the scores between protein groups (^∗^*p* < 0.05). (D) Enrichment of CVD risk protein interactors among hub proteins, DOX-correlated proteins, and DOX-correlated hub proteins. (E) Protein-protein interaction network for CVD proteins (square) and CVD protein interactors (circle) expressed within the co-expression network. Color represents if the protein is in a DOX-correlated module (red) or a non-DOX-correlated module (blue). Edges represent weighted correlation within the co-expression network. Node size indicates if a protein is a hub (large icon) or not a hub (small icon) protein. A CVD protein subnetwork containing the most highly connected proteins is presented here with the full CVD protein network displayed in Figure S16.

To understand how proteins in our network correspond to CVD more generally, we obtained GWAS data for 84 CVD traits available in the GWAS catalog. We assessed the relative likelihood for key network proteins to be contained in the set of proteins mapped to CVD (Figure 6A). CVD-associated proteins are neither enriched nor depleted among hub proteins, DOX-correlated proteins, or DOX-correlated hub proteins (Figure 6B), suggesting that they do not contribute to the key features of our protein network. These results are in line with the observation that DOX-correlated hub proteins are depleted for pQTLs and are intolerant to genetic mutation.

We next reasoned that proteins that are known to physically interact with CVD-associated proteins may provide a mechanism through which DNA damage associates with CVD risk. We therefore identified pairwise interactions between all 4,178 network proteins (STRINGdb; confidence score ≥0.9) and generated a total physical protein-protein interaction (PPI) network (*n* = 13,750 edges). We extracted the subnetwork containing CVD proteins (*n* = 213) and their interactors (*n* = 798; *n* = 909 edges; Data S2 & Table S4). We then asked whether CVD proteins and their interactors differ in their tolerance to mutation. CVD proteins have lower pLI scores than proteins they interact with (0.07 vs. 0.69; Wilcoxon rank-sum test; *p* < 0.05; Figure 6C). To test the robustness of these results, we analyzed 10,000 randomly generated subnetworks from the total network that maintained a similar degree distribution as the CVD protein network (See STAR Methods). The enrichment *p* value of CVD-associated proteins and their interactors is lower than the 5^th^ percentile of the random distribution (*p* = 0.00001 vs. *p* = 0.006), and the median pLI score difference between our test sets (0.63) is within the 95^th^ (0.64) and 96^th^ (0.62) percentiles of the randomly generated networks. These findings suggest that genes encoding CVD risk proteins are less likely to be essential compared to their interacting partners.

We next asked about the relative likelihood for hub proteins in the network to be contained in the set of CVD protein interactors. We first calculated the frequency of protein interactors falling in the same module. We find that the proportion of interacting proteins expressed in the same module is higher for interactions where one of the proteins is a hub protein compared to interactions that do not include a hub protein (Wilcoxon rank-sum test; *p* < 0.05; Figure S15). Although hub proteins are more likely to be co-expressed in the same module with their physical protein interactors, hub proteins are not enriched among CVD protein interactors (Figure 6D). However, CVD protein interactors are enriched for DOX-correlated proteins in comparison to proteins that are not DOX-correlated (Fisher’s exact test; OR = 1.5; CI = 1.1–2.1; *p* < 0.05; Figure 6D). We further find that CVD protein interactors are enriched for DOX-correlated hub proteins in comparison to hub proteins that are not DOX-correlated (OR = 5.1; CI = 1.8–15.1; *p* < 0.05; Figure 6D). We similarly observe a significant enrichment of CVD protein interactors among DOX-correlated hub proteins when identifying interactors with a lower stringency score (*n* = 49,020 interactions; STRINGdb confidence score ≥0.4; OR = 2.3; *p* < 0.05). These data suggest that the pathogenicity of CVD variants identified by GWASs might not only be a consequence of a direct effect on a protein’s function but also indirectly through interactions with essential proteins that are highly correlated to the DNA damage response in cardiomyocytes. We therefore illustrate the CVD protein and CVD protein interactor network together with our experimentally derived DOX-correlation and hub protein status annotations (Figures 6E and S16).

We posit that the DNA-damage-associated CVD protein network in cardiomyocytes can also be used to reduce the search space for druggable targets that might have the greatest impact on influencing various CVD phenotypes. We therefore also annotated each network protein by whether they are a target of an FDA-approved drug (Table S2).^33^ We identified 79 proteins in DOX-correlated modules that are druggable, including the chromatin-modifying enzymes HDAC1, HDAC2, and HDAC3. Seven of the druggable DOX-correlated proteins are CVD proteins (FADS1, FADS2, FINC, IGF1R, AT2B1, RL3, and TRXR1), and five have elevated expression in heart tissue (RYR2, ADT1, LDHB, ODO1, and AAPK2), thereby opening further avenues of investigation.

## Discussion

Many genetic loci have been associated with CVD. While the genes in these loci can be inferred to play a role in disease risk, the mechanisms behind these associations is often unclear. We hypothesized that a relevant environmental factor may provide insight. DNA damage is a ubiquitous stressor that is both implicated in and predictive of CVD.^3^ It can be induced through exogenous factors including through administration of drugs used in the treatment of cancer. To understand how DNA damage in cardiomyocytes influences CVD risk, we used an *in vitro* model of iPSC-CMs from multiple individuals to study the effects of DOX on the proteome. We constructed a co-expression network and identified many proteins that change in their abundance in response to DOX and characterized their network properties and tolerance to variation.

### DOX induces changes to the cardiomyocyte proteome relevant to anthracycline-induced cardiotoxicity

The effects of genetic variation and environmental perturbations on mediating risk for complex disease have most often been assayed through the transcriptome. This includes studies investigating the influence of DOX on cardiomyocytes.^24^^,^^27^^,^^28^ Here, we measured the effects of DOX on the cardiomyocyte proteome using both a pairwise differential abundance test and a co-expression network correlated with DOX treatment. We identified 319 differentially abundant proteins (8% of the proteome) and determined that 5 out of 12 co-expressed modules are correlated with DOX treatment. A previous study that assayed the proteomic response to anthracyclines, including DOX, using both human microtissues and tissues from heart failure patients identified four anthracycline-associated hub proteins.^51^ We find two of these proteins, BAG3 and hub protein CAND1, in DOX-correlated modules in cardiomyocytes, indicating the relevance of our cardiomyocyte model. In addition, our study generated protein data for thousands more proteins, implicating many more proteins in DOX toxicity. Conversely, we found little overlap between our data and DOX-treated rodent cardiac proteomic data from rat cardiomyocytes^52^ and rat heart tissue,^53^ suggesting species specificity in DOX responses.

Our network analysis identified the α module as the most DOX-correlated co-expression module. The 579 proteins in this module decrease in their abundance following DOX treatment and are enriched for proteins in gene regulatory processes and DNA damage repair. The most enriched processes relate to RNA processing and splicing. The associated proteins likely contribute to the large-scale splicing changes that have previously been identified following DOX treatment.^27^ Processes related to chromatin organization and histone modifications are also enriched, in line with previous work in a murine DOX toxicity model indicating effects on histone eviction and histone-modifying enzymes.^54^ This module is also enriched for transcription factors including GATA4, CTCF, ZNF629, ESSRA, GATAD2B, ZNF346, YBX3, GTF2I, and GATAD2A, indicating potential drivers of the gene expression changes observed due to DOX treatment in heart cells.^38^^,^^39^^,^^55^^,^^56^^,^^57^^,^^58^^,^^59^^,^^60^^,^^61^^,^^62^^,^^63^^,^^64^^,^^65^ Many of these processes are important across cell types, and we correspondingly observe decreased heart tissue specificity for proteins in this module. Indeed, DOX can lead to neurotoxicity, hepatotoxicity, and nephrotoxicity in addition to cardiotoxicity.^66^^,^^67^^,^^68^ Conversely the DOX-correlated δ module includes proteins with higher heart specificity than proteins in other modules and is enriched for processes related to mitochondrial functions such as oxidative phosphorylation. Proteins in this module may thus contribute to some of the *in vivo* tissue-specific effects of DOX on the heart.

The target of DOX is TOP2, expressed in the heart as both the α and β isoforms. It is TOP2B that is thought to mediate the cardiotoxic effects of anthracycline chemotherapeutics such as DOX. While we do not detect a change in TOP2B protein abundance in response to DOX, it is co-expressed in the ε module, which is DOX-correlated and consists of proteins downregulated in response to DOX. Notably, ∼75% of proteins shown to physically interact with TOP2B (10/13 expressed in iPSC-CMs), including CTCF, are present in DOX-correlated modules α and ε and exhibit high connectivity.^69^ GWASs have identified 10 risk loci associated with anthracycline-induced cardiotoxicity.^70^^,^^71^^,^^72^ We find three proteins encoded by genes mapped to these risk loci expressed in our network, where two are localized in the ε module. These proteins include POLRMT and RPL7. Decreased abundance of POLRMT, a DNA-directed RNA polymerase located in mitochondria, and RPL7, a component of the large ribosomal subunit, suggest that these proteins concordantly lead to decreased mitochondrial transcription and translation due to DOX treatment. Together, the proteins that we identify as responding to DOX in cardiomyocytes have been implicated in DOX-induced cardiotoxicity through molecular and genetic approaches.

While our study was designed to understand general principles of DOX treatment on cardiomyocytes using three individuals, our data suggest that there may be individual-specific effects on protein abundance and response to DOX and that studies including more individuals are warranted.

### DOX-induced protein expression changes are relevant to CVD

Our network approach allowed us to intersect sets of DOX-correlated proteins with proteins implicated in risk for complex CVDs. However, there are hundreds of proteins within each DOX-correlated module and hundreds of DAPs. Prioritization of proteins as diagnostic and therapeutic targets therefore remains a challenge. We provide annotations to subset module proteins by including DOX response effect size and direction, hub status, pLI score, druggability, and association with CVD, which may help in target selection for proteins most likely to modulate the cardiomyocyte response to DNA damage (Table S2). For example, the application of these filters uncovered highly responsive proteins that are central to the DOX response network, such as downregulated proteins GATA4, QKI, and TFAM, which have been shown to directly improve DOX-induced cardiotoxicity when their expression is increased.^39^^,^^73^^,^^74^ We highlight some proteins below that may contribute to a CVD trait and inform on cardioprotective drug development.

The α module contains proteins encoded by genes in loci associated with atrial fibrillation. These proteins include GATA4, LRRC10, RBM20, SLIT3, CAND2, GTF2I, HSPG2, FILIP1, MYO18B, CASZ1, and DPF3 that are downregulated in response to DOX treatment. Many of these proteins show evidence for independently impacting atrial fibrillation risk, as well as playing an essential role in cardiac development.^38^^,^^39^^,^^55^^,^^56^^,^^57^^,^^58^^,^^59^^,^^60^^,^^61^^,^^62^^,^^63^^,^^64^^,^^65^^,^^75^ The co-expression of these proteins involved in the genetic risk for atrial fibrillation indicates that DNA damage may mediate atrial fibrillation by collectively reducing the abundance of these critical proteins. Atrial fibrillation can be a cause or consequence of many complex CVD traits,^76^ highlighting the importance of these proteins to disease.

The β module, containing proteins with increased abundance due to DOX treatment, shows enrichment for both metabolic processes as well as processes involved in the translocation of enzymatic proteins to the nucleus, uniquely placing it at the intersection between the proteomic and metabolomic response to DNA damage. This includes the hub protein PRDX1, which has been shown to translocate to the nucleus upon DNA double-strand breakage and clear damage-induced nuclear reactive oxygen species and γH2AX.^77^ Similarly, four TCA cycle proteins help prevent DOX-mediated cellular damage by translocating from the mitochondria to the nucleus upon DNA damage.^78^ Three of these proteins, PDH-E1, MDH-2, and CS, are co-expressed in the β module. Therefore, the β module may identify proteins for future studies investigating how the nuclear translocation of enzymatic proteins can attenuate DOX-induced cardiotoxicity.

### Highly connected DNA-damage-associated proteins are intolerant to mutation

Our network analysis identified not only modules of co-expressed proteins but also subsets of highly connected hub proteins belonging to DOX-correlated and non-DOX-correlated modules. This allowed us to investigate the relationship between DNA-damage-associated connectivity and tolerance of these proteins to physiological and pathological variation. We find that DNA-damage-associated hub proteins are not only depleted for pQTLs but also enriched for loss-of-function-intolerant proteins, indicating the importance of these proteins. Considering the spectrum of connectivity, beyond the highly connected hub proteins, revealed increasing pLI values with increasing protein connectivity for DOX-correlated module proteins. pLI values remain low across a range of connectivities for non-DOX-correlated module proteins. The trend for DOX-correlated proteins was observable in both total connectivity and intra-modular connectivity, but not inter-modular connectivity, suggesting that intolerance to mutation in the network is centered around DOX-correlated modules and their related biological processes. The opposite trend is true for pQTLs, where enrichment tends to decrease with increased connectivity of DOX-correlated proteins. It has previously been shown that pQTLs are depleted among hub proteins in a steady-state network.^43^ Here, we show that DOX-correlated hub proteins are depleted for *cis-* and *trans*-pQTLs compared to non-DOX-correlated hub proteins, and those that are pQTLs have lower effect sizes in the DOX-correlated set. These results suggest that the proteins most central to the DNA damage response are constrained in their expression with little variation across individuals and support the observation that these proteins are intolerant to loss-of-function mutations. We note that the set of unassigned proteins are neither enriched nor depleted in mutation-intolerant genes or pQTLs, suggesting that as a group these do not have distinct genetic tolerance properties. The differential relationship between connectivity, genetic influence, and pLI emphasizes the evolutionary constraint of DOX-correlated hub proteins by purifying selection to minimize potential disruptions in their expression.

### Highly connected DNA-damage-associated proteins influence CVD risk proteins through protein-protein interactions

While CVD risk proteins are not enriched among hub proteins or DOX-correlated proteins, physical protein interactors of CVD risk proteins are enriched among DOX-correlated proteins and DOX-correlated hub proteins in particular. This suggests that DNA damage can affect the expression of proteins that interact with proteins implicated in CVD risk, hinting at the mechanism through which some of the disease-associated loci might exert their effects. For example, DOX-correlated hub proteins PBRM1 and SMARCC1 are both chromatin regulators that interact with atrial-fibrillation-associated protein DPF3.^65^ These findings are also corroborated by the observation that GWAS risk proteins are generally very tolerant to mutation, but their physical PPIs are not. However, there are exceptions to this trend such as is observed in atrial fibrillation, where GATA4, GTF2I, and CASZ1 are both CVD proteins and DOX-correlated hub proteins. These data support the notion that genetic variation could contribute to CVD phenotypes by altering the stability and functionality of regulatory proteins that are central to the proteomic DNA damage response network through physical protein interactions. Therefore, our network pinpoints CVD risk proteins that are highly connected to proteins central to the cardiomyocyte DNA damage response that can be prioritized for cell-type-specific co-immunoprecipitation studies that have proved informative for understanding mechanisms behind genes implicated in coronary artery disease and autism spectrum disorder.^22^^,^^23^

### Considering the DNA damage response network in the context of the omnigenic model

The omnigenic model for complex phenotypes posits that thousands of genes expressed in disease-relevant cell types can influence complex traits, whereby core genes have a direct impact on the phenotype, while peripheral genes influence it indirectly through distant gene interactions.^79^ In an omnigenic architecture, the majority of the heritability influencing complex traits with polygenic architecture is distributed across peripheral genes and involves extensive regulatory networks connecting them to core genes.^79^^,^^80^ In practice, core genes are likely identified on a graded scale rather than a binary classification, where heritability decreases as the degree of separation from core genes is reduced. The use of co-expression networks to investigate the omnigenic model provides a powerful approach to untangle the complex interactions specified in this framework.

Co-expression networks across different conditions or tissues can use connectivity as a method to identify core and peripheral genes and specify the distribution of heritability across degrees of separation. For example, Mähler et al., used a transcriptional co-expression network from *Populus tremula* leaf buds to demonstrate that eQTL genes are predominantly located at the network’s periphery and that connectivity is inversely correlated with eQTL effect sizes, implying that core genes (hub genes) within modules experience strong selective pressures.^81^ The purifying selection predominantly acting on core genes implies an evolutionary conservation that possibly underscores their fundamental biological roles. This finding is also consistent with the notion that damage to core genes by loss-of-function mutations tends to have strong effects on disease risk.^79^^,^^81^ Fóthi et al. evaluated the omnigenic model’s application to autism spectrum disorder using brain-specific gene interaction networks and found that autism gene clusters are significantly more connected to each other and the peripheral genes in brain-related tissues than in non-brain-related tissues.^82^ These data support the notion that disease-relevant tissues are the appropriate context for assessing omnigenic architectures to better understand complex traits. Hartl et al. used this foundation to generate a co-expression network derived from gene expression profiles across 12 brain regions to contextualize the functional pathways of risk genes for multiple neuropsychiatric diseases.^18^ Despite the omnigenic model’s suggestion that disease risk is mediated by a small number of core genes indirectly influenced by peripheral genes, the study indicated that gene effects are distributed more continuously across the networks rather than being separated into distinct core and peripheral categories.

The omnigenic model suggests that the connections between peripheral and core genes vary by trait and include transcriptional networks, post-translational modifications, and protein-protein interactions^79^^,^^83^ and that genetic variants influencing disease may affect expression in specific cell types or conditions.^79^ The aforementioned studies all utilize steady-state expression data to construct their networks, whereas an omnigenic architecture relevant to disease may emerge under the conditions of specific cell stressors that drive the purifying selection pressure that core genes are placed under. The results obtained for DOX-correlated modules resemble what would be predicted by an omnigenic architecture, where there is a negative correlation between the influence of genetic variation and network connectivity. However, the opposite is true for proteins that are not correlated to DOX. This suggests that the core-periphery structure may develop in response to selective pressures. Our dynamic network differentiates proteins that are actively responding to stress from those that are not. While modules derived from steady-state networks can enrich for various biological processes, the connectivity observed at steady state may not accurately reflect gene relationships under disease-relevant conditions. Instead, the core-periphery structure may be dynamic, shifting according to different cellular states. If true, it means that networks constructed in response to various stressors might establish a connectivity profile that pinpoints unique or shared core-peripheral relationships to different cell states. Therefore, networks generated from cell models that capture the dynamic molecular responses to selective pressures or disease-associated stimuli may be needed to more accurately understand how the core-periphery structure described in the omnigenic model emerges in the transition from non-disease to disease states.

In summary, there are no studies integrating proteomic data from cardiomyocytes subjected to DNA damage with measures of genetic tolerance to variation and disease. Here, we profiled protein abundance in cardiomyocytes treated with DOX across multiple individuals. We found that the level of protein connectivity in DNA-damage-associated co-expression modules influences the tolerance to genetic variation. We believe that the data and analysis presented here will be a resource for further studies into the mechanistic effects of DNA damage on the cardiomyocyte proteome and DOX-induced cardiotoxicity, as well as for studies investigating the architecture of complex traits in response to perturbation.

### Limitations of the study

We generate cardiomyocytes through directed differentiation of iPSCs as it allows us to include multiple individuals and use these cardiomyocytes in carefully controlled experiments where we can treat the same batch of cells with DOX and measure their protein abundance. However, it is possible that our *in vitro* system may not fully recapitulate the *in vivo* molecular profile. We also selected a single, sub-lethal, dose of DOX to study the primary effects of DNA damage on the proteome. It is possible that different doses of DOX would apply different selective pressures on the proteome and identify different response proteins and mutation tolerance. However, we believe our results using a clinically relevant dose of a widely used chemotherapeutic agent allow us to provide useful insights. Similarly, the response to DNA damage may be temporally dynamic and hence our results may not extrapolate to shorter or longer exposure times.

While mass spectrometry is a powerful tool for measuring protein abundance, it has inherent limitations that can affect the comprehensiveness and accuracy of the data acquired. For example, it may not detect low-abundance proteins effectively, leading to an incomplete representation of the proteome. Further, while protein abundance is a critical metric, it is not the only relevant measure when assessing protein function and cellular responses. Other important protein metrics include post-translational modifications such as phosphorylation, ubiquitination, and glycosylation, which can significantly alter protein function, stability, localization, and interactions. Future studies that integrate more comprehensive transcriptional and proteomic networks that capture multiple time points and greater protein coverage may enhance the findings from similarly designed studies.

## Resource availability

### Lead contact

Requests for further information, resources, and reagents should be directed to and will be fulfilled by the lead contact, Dr. Michelle Claire Ward (miward@utmb.edu).

### Materials availability

This study did not generate new unique reagents.

### Data and code availability

• Data: mass spectrometry data generated in this study are available in the MassIVE database (https://massive.ucsd.edu/ProteoSAFe/static/massive.jsp). The accession number is listed in the key resources table.
• Code: all original code has been deposited at GitHub and is publicly available as of the date of publication. The link is listed in the key resources table. Custom analysis scripts were generated using workflowr.^84^
• Other items: any additional information required to reanalyze the data reported in this paper is available from the lead contact upon request.

## Acknowledgments

We thank all members of the Ward Lab for helpful discussions. We thank Dr. Kelly A. Frazer and the University of California San Diego for providing the iPSC lines through the iPSCORE resource. We acknowledge the Wellcome Trust Sanger Institute as the source of the WTSIi048-A human induced pluripotent cell line, which was generated under the human induced pluripotent stem cell initiative funded by a grant from the Wellcome Trust and Medical Research Council, supported by the 10.13039/100010269Wellcome Trust (WT098051) and the NIHR/Wellcome Trust Clinical Research Facility, and acknowledge Life Science Technologies Corporation as the provider of Cytotune. We thank the Mass Spectrometry Facility at the University of Texas Medical Branch for performing the mass spectrometry experiments, and the Flow Cytometry and Cell Sorting Core Facility for access to flow cytometers. This work was funded by a 10.13039/100004917Cancer Prevention & Research Institute of Texas (https://www.cprit.state.tx.us/) Recruitment of First-Time Faculty Award (RR190110) to M.C.W., the 10.13039/100000002National Institutes of Health grant R35GM150459 to M.C.W., and CPRIT grant RP190682 to W.K.R. in partial support of the UTMB Mass Spectrometry Facility. O.D.J. was supported by a Jeane B. Kempner Predoctoral Fellowship administered through UTMB (http://www.kempnerfund.org/).

## Author contributions

M.C.W. conceived and designed the study. S.P., J.A.G., and W.K.R. performed the experiments and generated the data. O.D.J., S.P., and M.C.W. analyzed the data. O.D.J. and S.P. visualized the data. O.D.J. and M.C.W. wrote the paper with input from all authors. M.C.W. supervised the project. All authors read and approved the final manuscript.

## Declaration of interests

The authors declare no competing interests.

## STAR★Methods

### Key resources table

**Table:** 

REAGENT or RESOURCE	SOURCE	IDENTIFIER
**Antibodies**		

Anti-Cardiac Troponin T antibody	BD Biosciences	Cat# 564767; RRID: AB_2738939
Phospho-Histone H2A.X (Ser139) Rabbit mAb	Thermo Fisher Scientific	Cat# NC1602516; RRID: AB_2898141
Donkey anti-Rabbit Alexa Fluor 594 antibody	Invitrogen	Cat# A-21207; RRID: AB_141637

**Chemicals, peptides, and recombinant proteins**		

mTESR1	Stem Cell Technology	Catalog No. 85850
Penicillin/Streptomycin	Corning	Catalog No. 30-002-Cl
Matrigel hESC-qualified Matrix	Corning	Catalog No. 354277
CHIR99021 trihydrochloride	Tocris Bioscience	Catalog No. 4953
RPMI 1640	Corning	Catalog No. 15-040-CM
B-27 minus insulin	Thermo Fisher Scientific	Catalog No. A1895601
GlutaMAX	Thermo Fisher Scientific	Catalog No. 35050-061
Wnt-C59	Tocris Bioscience	Catalog No. 5148
RPMI without glucose	Thermo Fisher Scientific	Catalog No. 11879
L-Ascorbic acid 2-phosphate sesquimagnesium salt	Santa Cruz Biotechnology	Catalog No. sc228390
Human Recombinant Albumin	Sigma-Aldrich	Catalog No. A0237
HEPES (L(+)Lactic acid sodium	Sigma-Aldrich	Catalog No. L7022
Trypsin-EDTA	Corning	Catalog No. 25–053 Cl
DMEM without glucose	Thermo Fisher Scientific	Catalog No. A1443001
FBS	Genemate	Catalog No. S1200-500
Galactose	Sigma-Aldrich	Catalog No. G5388
Sodium pyruvate	Thermo Fisher Scientific	Catalog No. 11360–070
FOXP3/Transcription Factor Staining Buffer Set	Thermo Fisher Scientific	Catalog No. 00–5523
autoMACS® Buffer	Miltenyi Biotec	Catalog No. 130-091-221
Doxorubicin	Sigma-Aldrich	Catalog No. D1515
Hoechst 33342 nucleic acid stain	Thermo Fisher Scientific	Catalog No. PI62249
Tris(2-carboxyethyl) phosphine	Thermo Fisher Scientific	Catalog No. 77720
Trypsin	Promega	Catalog No. V5280

**Critical commercial assays**		

Zombie Violet Fixable Viability Kit	BioLegend	Catalog No. 423113
BCA Protein Assay kit	Thermo Fisher Scientific	Catalog No. 23227
Nanoflow liquid chromatography-tandem mass spectrometry and analysis	UltiMate 3000 RSLCnano, Dionex	N/A
Quantitative Fluorometric Peptide Assay	ThermoFisher Scientific	Catalog No. 23290

**Deposited data**		

Mass Spectrometry data	MassIVE database (https://massive.ucsd.edu/ProteoSAFe/static/massive.jsp)	MassIVE: MSV000094446
Code	GitHub	GitHub: https://github.com/mward-lab/Johnson_DNA_damage_DOX_2025

**Experimental models: Cell lines**		

Human iPSCORE iPSC line: UCSD143i-87-1	University of California San Diego & the WiCell Research Institute	https://www.wicell.org/home/stem-cells/catalog-of-stem-cell-lines/ucsd143i-87-1.cmsx?closable=true
Human iPSCORE iPSC line: UCSD131i-77-1	University of California San Diego & the WiCell Research Institute	https://www.wicell.org/home/stem-cells/catalog-of-stem-cell-lines/ucsd131i-77-1.cmsx?closable=true
Human HipSci iPSC line: WTSIi048-A	EBiSC & ECACC	https://www.ebi.ac.uk/ena/browser/view/ERZ368931?show=analyses

**Software and algorithms**		

MSConvert	Command line software	Version 3
DIA-NN	Github	Version 1.9
ImageJ	National Inst. Of Health	Version 1.54i
Cytoscape	Open source	Version 3.10.0
AnnotationDbi	R package	Version 1.66.0
biomaRt	R package	Version 2.60.1
BioNERO	R package	Version 1.12.0
clusterProfiler	R package	Version 4.12.6
dynamicTreeCut	R package	Version 1.63–1
edgeR	R package	Version 4.2.1
ggraph	R package	Version 2.2.1
igraph	R package	Version 2.0.3
impute	R package	Version 5.1–3
limma	R package	Version 3.60.4
org.Hs.e.g.,.db	R package	Version 3.19.1
pheatmap	R package	Version 1.0.12
ruv	R package	Version 0.9.7.1
stats	R package	Version 4.4.1
SummarizedExperiment	R package	Version 1.34.0
tidygraph	R package	Version 1.3.1
WGCNA	R package	Version 1.72–5
Proteome Discoverer	Thermo Fisher Scientific	Version 2.2.0388

**Other**		

LSRFortessa Cell Analyzer	BD Bioscience	https://www.bdbiosciences.com/en-us/products/instruments/flow-cytometers/research-cell-analyzers/bd-lsrfortessa
nano-LC chromatography system	Thermo Fisher Scientific (UltiMate 3000 RSLCnano, Dionex)	https://www.thermofisher.com/order/catalog/product/ULTIM3000RSLCNANO
Orbitrap Eclipse Tribrid Mass Spectrometer	Thermo Fisher Scientific	https://www.thermofisher.com/us/en/home/industrial/mass-spectrometry/liquid-chromatography-mass-spectrometry-lc-ms/lc-ms-systems/orbitrap-lc-ms/orbitrap-tribrid-mass-spectrometers/orbitrap-eclipse-tribrid-mass-spectrometer.html

### Experimental model and study participant details

#### Human induced pluripotent stem cell (iPSC) lines

Three human iPSC lines were used in this study. All lines were derived from skin fibroblasts of healthy female donors with no prior history of cardiac disease.

(1) Individual 1 (UCSD131i-77-1): An iPSC line derived from a 21-year-old Asian-Chinese female donor that is part of the iPSCORE collection at the University of California San Diego. The cell line was generated by Dr. Kelly A. Frazer as part of the National Heart, Lung and Blood Institute Next Generation Sequencing Consortium^85^ and is available through the biorepository at WiCell Research Institute (Madison, WI, USA), or through contacting Dr. Kelly A. Frazer at the University of California, San Diego.
(2) Individual 2 (UCSD143i-87-1): An iPSC line derived from a 23-year-old Asian-Chinese female donor that is part of the iPSCORE collection at the University of California San Diego. The cell line was generated by Dr. Kelly A. Frazer as part of the National Heart, Lung and Blood Institute Next Generation Sequencing Consortium^85^ and is available through the biorepository at WiCell Research Institute (Madison, WI, USA), or through contacting Dr. Kelly A. Frazer at the University of California, San Diego.
(3) Individual 3 (WTSIi048-A): An iPSC line derived from a 72-year-old White British female donor that is part of the HipSci project, funded by the Wellcome Trust and Medical Research Council.^86^ The cell line is available from the European Bank of induced pluripotent Stem Cells (EBiSC) and the European Collection of Authenticated Cultures (ECACC).

All iPSC lines were maintained in feeder-free culture conditions with mTeSR1 medium on Matrigel-coated plates. Cells were cultured at 37°C in 5% CO_2_ and 95% humidity and routinely passaged using EDTA-based dissociation. All iPSC lines were tested and confirmed negative for mycoplasma contamination prior to experimentation.

#### Ethical approvals and consent

(1) The iPSCORE lines (UCSD131i-77-1 and UCSD143i-87-1) were generated with informed written consent from donors and approval by the Institutional Review Boards of the University of California San Diego and The Salk Institute (IRB protocol: 110776ZF).
(2) The HipSci line (WTSIi048-A) was generated with donor consent and approved by the East of England - Cambridge Central Research Ethics Committee (REC 15/EE/0049).

#### Sex and gender consideration

This study used iPSC lines derived from female donors. As such, the influence of biological sex on gene expression and phenotype could not be evaluated across sexes. This represents a limitation in terms of generalizability of our findings to male-derived cardiomyocytes. Future studies including male donor-derived iPSC lines are needed to address potential sex-specific effects in the observed phenotypes.

#### Sample size and allocation to experimental groups

This study included three human iPSC lines from three donors. The sample size was determined based on proteomics benchmarks and established molecular responses to DOX. Cardiomyocytes from each individual were treated with DOX or Vehicle. The treatment was replicated three times for Individual 3, allowing for modeling of technical variation in the experimental process. 10 samples in total were generated. Cardiomyocytes from each individual were randomly assigned to the DOX or Vehicle treatment groups. Protein was extracted from all samples in one batch.

### Method details

#### iPSC culture

Feeder-independent iPSCs were cultured in mTESR1 (85850, Stem Cell Technology, Vancouver, BC, Canada) media with 1% Penicillin/Streptomycin (30-002-Cl, Corning, Bedford, MA. USA) on hESC-qualified Matrigel Matrix (354277, Corning, Bedford, MA, USA) at a dilution of 1:100. iPSCs were passaged with dissociation reagent (0.5 mM EDTA, 300 mm NaCl in PBS) when they attained 70–80% confluency, approximately every 3–5 days.

#### Cardiomyocyte differentiation

Cardiomyocyte differentiations were performed as previously described.^26^ Briefly, iPSC lines were seeded in Matrigel-coated culture dishes (Days −6/-5) and cultured until 85–90% confluent. Differentiations were initiated (Day 0) by adding 12 μM of the GSK3 inhibitor, CHIR99021 trihydrochloride (4953, Tocris Bioscience, Bristol, UK) in Cardiomyocyte Differentiation Media (CDM) [500 mL RPMI 1640 (15-040-CM, Corning), 10 mL B-27 minus insulin (A1895601, ThermoFisher Scientific, Waltham, MA, USA), 5 mL GlutaMAX (35050-061, ThermoFisher Scientific), and 5 mL of Penicillin/Streptomycin (100X) (30-002-Cl, Corning)]. After 24 h (Day 1), the media was replaced with fresh CDM. On Day 3 (after 48 h) media was replaced with CDM containing 2 μM of the Wnt signaling inhibitor Wnt-C59 (5148, Tocris Bioscience) in CDM. CDM was replaced on Day 5, 7, 10 and 12. We observed spontaneously beating cells between Day 7–10. iPSC-CMs were purified by metabolic selection with glucose-free, lactate-containing media [500 mL RPMI without glucose (11879, ThermoFisher Scientific), 106.5 mg L-Ascorbic acid 2-phosphate sesquimagnesium salt (sc228390, Santa Cruz Biotechnology, Santa Cruz, CA, USA), 3.33 mL 75 mg/mL Human Recombinant Albumin (A0237, Sigma-Aldrich, St Louis, MO, USA), 2.5 mL 1 M lactate in 1 M HEPES (L(+)Lactic acid sodium (L7022, Sigma-Aldrich)), and 5 mL Penicillin/Streptomycin] added on Day 14, 16 and 18. On Day 20, iPSC-CMs were detached with 0.05% Trypsin-EDTA solution (25–053 Cl, Corning), and a single cell suspension was generated by straining. iPSC-CMs were counted with a Countess 2 machine. 1.5 million iPSC-CMs were plated per well of a 0.1% Gelatin-coated 6-well plate in 3 mL Cardiomyocyte Maintenance Media (CMM) [500 mL DMEM without glucose (A1443001, ThermoFisher Scientific), 50 mL FBS (S1200-500, Genemate), 990 mg Galactose (G5388, Sigma-Aldrich), 5 mL 100 mM sodium pyruvate (11360–070, ThermoFisher Scientific), 2.5 mL 1 M HEPES (SH3023701, ThermoFisher Scientific), 5 mL Glutamax (35050–061, ThermoFisher Scientific), 5 mL Penicillin/Streptomycin]. The iPSC-CMs were matured in culture for 10 days, with CMM replaced on Day 23, 25, 27, 28, and 30.

#### iPSC-CM purity determination using flow cytometry

After differentiation of iPSCs from Individuals 2 & 3, the iPSC-CM purity was determined using flow cytometry. Between Day 25–27 of differentiation the iPSC-CMs were dissociated with 0.05% Trypsin-EDTA solution and strained to generate a single cell suspension. One million cells were stained with Zombie Violet Fixable Viability Kit (423113, BioLegend) for 30 min at 4°C prior to fixation and permeabilization (FOXP3/Transcription Factor Staining Buffer Set, 00–5523, ThermoFisher Scientific) for 30 min at 4°C. Cells were stained with 5 mL PE Mouse Anti-Cardiac Troponin T antibody (564767, clone 13–11, BD Biosciences, San Jose, CA, USA) for 45 min at 4°C. Cells were washed three times in permeabilization buffer and re-suspended in autoMACS Running Buffer (130-091-221, Miltenyi Biotec, Bergisch Gladbach, Germany). We used several negative controls in each flow cytometry experiment: 1) iPSCs, which should not express TNNT2, 2) an iPSC-CM sample that has not been labeled with viability stain or TNNT2 antibody, 3) an iPSC-CM sample that is only labeled with the viability stain and 4) an iPSC-CM sample that is only labeled with TNNT2. 10,000 cells were captured and profiled on the BD LSRFortessa Cell Analyzer. Multiple gating steps were performed to determine the proportion of TNNT2-positive cells: 1) Cellular debris was removed by gating out cells with low granularity on FSC versus SSC density plots, 2) From this population, live cells were identified as the violet laser-excitable, Pacific Blue dye-negative population, 3) TNNT2-positive cells were identified within the set of live cells and any cells that overlap the profiles of the negative control samples were excluded. iPSC-CM purity is reported as the proportion of TNNT2-positive live cells.

#### Drug treatment of iPSC-CMs

On Day 29, iPSC-CMs were treated with 0.5 μM of Doxorubicin (D1515, Sigma-Aldrich) or vehicle (Molecular Biology grade water) in fresh CMM media for 24 h. The treatment for Individual 3 was replicated two additional times yielding 10 samples in total across three individuals. Post-treatment, cells were washed twice and scraped in ice-cold PBS. iPSC-CMs were flash-frozen and stored at −80°C prior to further processing.

#### γH2AX immunofluorescence staining and quantification of DNA double-strand breaks

300,000 iPSC-CMs were seeded per well of a 24-well plate in CMM media. Cells were treated with 0.5 μM DOX or vehicle (DMSO). The treated cells were fixed in 4% paraformaldehyde for 15 min and permeabilized with 0.25% DPBS-T for 10 min at room temperature. Cells were incubated with 5% BSA:DPBS-T for 30 min at room temperature, then incubated with a 1:500 dilution of γ-H2AX primary antibody in BSA:DPBS-T overnight at 4°C (Phospho-Histone H2A.X (Ser139) Rabbit mAb; NC1602516; Fisher Scientific). Cells were incubated with Fluorochrome-conjugated secondary antibody (Donkey anti-Rabbit Alexa Fluor 594, A-21207, Invitrogen) for 1 h at room temperature at a 1:1000 dilution in DPBS-T. Cell nuclei were counterstained with Hoechst 33342 nucleic acid stain (PI62249, Thermo Scientific) for 10 min in the dark. Stained cells were subjected to fluorescence microscopy. The total number of nuclei and γH2AX-positive nuclei were quantified using the cell counter plugin of ImageJ software.^87^ The number of γH2AX-positive nuclei were divided by the total number of nuclei to determine the percentage of γH2AX-positive nuclei in DOX- and vehicle-treated iPSC-CMs for three different individuals. The percentage of γH2AX-positive cells between vehicle- and DOX-treated iPSC-CM samples was compared by t test.

#### Protein isolation and quantification

Protein was isolated from iPSC-CMs by lysing the cells with RIPA buffer [1.5 mL 5 M NaCl, 1 mL Triton X-100, 1 g Na deoxycholate, 1 mL 10% SDS, 1 mL 1 M Tris pH 7.4 and 45 mL dH_2_O] with protease inhibitor for 1 h at 4°C. Isolated proteins were quantified by using the BCA Protein Assay kit (23227, Thermo Scientific) according to the manufacturer’s instructions.

#### Protein digestion

The samples were prepared similarly as previously described.^88^ Briefly, 15 μg of protein were solubilized with 60 μL of 50 mM Triethylammonium bicarbonate (TEAB) pH 7.55. The proteins were then reduced with 10 mM Tris(2-carboxyethyl) phosphine (TCEP) (77720, Thermo) and incubated at 65°C for 10 min. The sample was then cooled to room temperature and 1 μL of 500 mM iodoacetamide acid was added and allowed to react for 30 min in the dark. Then, 3.3 μL of 12% phosphoric acid was added to the protein solution followed by 200 μL of binding buffer (90% Methanol, 100mM TEAB pH 8.5). The resulting solution was added to S-Trap spin column (protifi.com) and passed through the column using a bench top centrifuge (60 s spin at 1,000 g). The spin column is washed with 150 μL of binding buffer and centrifuged. This is repeated two times. 30 μL of 20 ng/μL Trypsin is added to the protein mixture in 50 mM TEAB pH 8.5, and incubated at 37°C overnight. Peptides were eluted twice with 75 μL of 50% acetonitrile, 0.1% formic acid. Aliquots of 20 μL of eluted peptides were quantified using the Quantitative Fluorometric Peptide Assay (Pierce, Thermo Fisher Scientific). Eluted volume of peptides corresponding to 5.5 μg of peptides are dried in a speed vac and resuspended in 27.5 μL 1.67% acetonitrile, 0.08% formic acid, 0.83% acetic acid, 97.42% water and placed in an autosampler vial.

#### Nanoflow liquid chromatography mass spectrometry

Peptide mixtures were analyzed by nanoflow liquid chromatography-tandem mass spectrometry (nanoLC-MS/MS) using a nano-LC chromatography system (UltiMate 3000 RSLCnano, Dionex), coupled on-line to a Thermo Orbitrap Eclipse mass spectrometer (Thermo Fisher Scientific, San Jose, CA) through a nanospray ion source. Instrument performance was verified by analyzing a standard six protein mix digest before the sample set run, between each experimental block and at the end of the experiment. The six protein mix data files were analyzed to confirm that instrument performance remained consistent throughout the experiment. A direct injection method using 3 μL of digest onto an analytical column was used; Aurora (75 μm × 25 cm, 1.6 μm) from (IonOpticks). After equilibrating the column in 98% solvent A (0.1% formic acid in water) and 2% solvent B (0.1% formic acid in acetonitrile (ACN)), the samples (2 μL in solvent A) were injected (300 nL/min) by gradient elution onto the C18 column as follows: isocratic at 2% B, 0–5 min; 2%–6%, 5–5.1 min; 6%–25% 5.1–105 min, 25%–50% B, 105–120 min; 50%–90% B, 120–122 min; isocratic at 90% B, 122–124 min; 90%–5%, 124–125 min; isocratic at 5% B, 125–126 min; 5%–90% 126–128 min; isocratic for 1 min; 90%–2%, 129–130 min; and isocratic at 2% B, till 150 min.

#### NanoLC MS/MS analysis for DDA

All data were acquired using an Orbitrap Eclipse in positive ion mode using a top speed data-dependent acquisition (DDA) method with a 3 s cycle time and a spray voltage of 1600 V. The survey scans (m/z 375–2000) were acquired in the Orbitrap at 120,000 resolution (at m/z = 400) in profile mode, with a maximum injection time of 100 ms and an AGC target of 1,000,000 ions. The S-lens RF level was set to 30. Isolation was performed in the quadrupole with a 1.6 Da isolation window, and HCD MS/MS acquisition was performed in profile mode using the orbitrap at a resolution of 15,000 using the following settings: parent threshold = 5,000; collision energy = 30%; AGC target at 125,000 using the default settings. Monoisotopic precursor selection (MIPS) and charge state filtering were on, with charge states 2–10 included. Dynamic exclusion was used to remove selected precursor ions, with a +/− 10 ppm mass tolerance, for 30 s after acquisition of one MS/MS spectrum.

#### NanoLC MS/MS analysis for DIA

All LC-MS/MS data were acquired using an Orbitrap Eclipse in positive ion mode using a data-independent acquisition (DIA) method with 16 Da windows from 400 to 1000 and a loop time of 3 s. The survey scans (m/z 350–1500) were acquired in the Orbitrap at 60,000 resolution (at m/z = 400) in centroid mode, with a maximum injection time of 118 ms and an AGC target of 100,000 ions. The S-lens RF level was set to 60. Isolation was performed in the quadrupole, and HCD MS/MS acquisition was performed in profile mode using the orbitrap at a resolution of 30000 using the following settings: collision energy = 33%, IT 54 ms, AGC target = 50,000. A pooled sample was used to create spectral libraries that we search the individual samples against by injecting 5 times using narrow (4 Da), staggered windows over 100 m/z ranges from 400 to 900 m/z in a technique called gas phase fractionation as described in Searle et al.^89^

#### Database searching for DDA proteins

Tandem mass spectra were extracted and charge state deconvoluted using Proteome Discoverer (Thermo Fisher, version 2.2.0388). Deisotoping was not performed. All MS/MS spectra were searched against a Uniprot Human database using Sequest and the Minora node used to perform Label-Free Quan (LFQ) using the MS peak areas for each of the peptide-spectral matches (PSMs). Searches were performed with a parent ion tolerance of 5 ppm and a fragment ion tolerance of 0.02 Da. Trypsin was specified as the enzyme, allowing for two missed cleavages. Fixed modification of carbamidomethyl (C) and variable modifications of oxidation (M) and deamidation were specified in Sequest. Protein identities reported at 1% false discovery rate were considered for filtering and downstream analysis.

#### Database searching for DIA proteins

The raw data was demultiplexed to mzML with 10 ppm accuracy after peak picking in MSConvert.^90^ The resulting mzML files were searched in MSFragger^91^ and quantified via DIA-NN^92^ using the following settings: peptide length range 7–50, protease set to Trypsin, 2 missed cleavages, 3 variable modifications, clip N-term M on, fixed C carbamidomethylation, variable modifications of methionine oxidation and n-terminal acetylation, MS1 and MS2 accuracy set to 20 ppm, 1% FDR, and DIANN quantification strategy set to Robust LC (high accuracy). The files were searched against a database of human acquired from Uniprot (18^th^ December, 2023). The gas-phase fractions were used only to generate the spectral library, which was used for analysis of the individual samples.

#### Abundance matrix filtering and imputation

We removed four non-human or uncharacterized proteins (UniProt: Q6ZSR9, P15252, P25691, P00761). Proteins that were missing across 50% or more of the samples were removed. For the remaining 246 proteins, missing values were imputed using k-Nearest Neighbors from the impute package knn. with, parameters k (number of neighbors to be used in the imputation) and rowmax (the maximum percent missing data allowed in any row).^30^ Using k = 10 in the impute.knn function leverages all available samples to impute missing values, given the small sample size of our dataset. The rowmax = 0.4 parameter allows for imputing rows with up to 40% missing values, balancing the need to retain as much data as possible while maintaining the quality of the imputations. These steps led to a total of 4,178 analyzable proteins across all samples.

#### Removal of unwanted technical variation and normalization

To eliminate unwanted technical variation from the log_2_-transformed imputed abundance matrix, we adjusted the 10 sample data matrix using both negative controls and the replicate data with the RUV-III function in the ruv R package.^93^ Negative control proteins were defined as the 5% least variable proteins across all samples. The RUV-corrected abundance matrix and RUV factors were used in downstream analysis.

#### Comparison of the iPSC-CM proteome with the proteome across human tissues

Protein abundance values across 26 tissues was obtained from Jiang et al.^20^ We identified the set of proteins expressed across the 26 tissues and our iPSC-CMs. We calculated the median abundance across individuals for the set of 26 tissues, as well as for our iPSC-CMs. The median abundance between our iPSC-CMs and each of the 26 tissues was correlated using Pearson correlation.

#### WGCNA network construction

We adopted the Weighted Gene Co-expression Network Analysis (WGCNA) methodology to investigate correlations between protein abundances in our RUV-corrected abundance matrix of DOX- and VEH-treated iPSC-CMs.^32^ The WGCNA framework, primarily utilizing wrapper functions from BioNERO, was implemented for this analysis.^94^ We first established a scale-free network topology, achieved by determining the appropriate soft threshold power using the SFT_fit function. After iterating different soft power thresholds (β), the linear regression of log_10_(k) versus log_10_(p(k)) indicates that by setting β = 20, the network is close to a scale-free network, where k is the whole network connectivity and p(k) is the corresponding frequency distribution. This setting resulted in our network attaining a scale-free fit index of 0.71 (quantification of how well the network approximates a scale-free topology), with a mean node connectivity of 65.4 and median connectivity of 53.6. We identified modules of co-expressed proteins using a modified workflow as previously described.^32^^,^^94^ The workflow was encapsulated in the exp2gcn2 function from the BioNERO package. We selected a signed network with a soft power threshold of 20, merging threshold of 0.85, and the pearson correlation method. We computed the Topological Overlap Matrix (TOM), a measure of network connectivity that emphasizes the shared neighbors between protein pairs to enhance the robustness and reliability of the calculated adjacency network. Using hierarchical clustering on the dissimilarity TOM (dissTOM), we identified initial modules of co-expressed proteins. The dynamic tree cut method using the cutreeDynamicTree function with maxTreeHeight of 3, minimum module sizes of 40 and no deep splitting was applied to the protein dendrogram to define these modules by modifying the exp2gcn2 function. The module eigenproteins (MEs), representing the first principal component for the module, were then calculated across modules with moduleEigengenes. These eigenproteins served as characteristic expression profiles of proteins within a module and were used to assess the interrelation between modules. Similar modules were merged based on the eigenprotein dissimilarity, ensuring that highly correlated modules were combined. Modules whose eigenproteins had a correlation of 0.85 or greater were merged.

We used three types of connectivity metrics to describe how each node/protein in the network related to other nodes/proteins. Total connectivity was calculated by summing the weighted correlations between each protein and all other proteins in the network (kTotal). Intra-modular connectivity (kIN) was calculated by summing the weighted correlations between each protein and all other proteins in the assigned module. Extra-modular connectivity (kOut) was calculated by summing the weighted correlations between each protein and all other proteins outside the assigned module.

#### Identification of hub proteins

We identified hub proteins that might play central roles in the biological processes represented by each module. We used the get_hubs_gcn function to identify hub proteins within our protein abundance correlation network. Hub proteins within modules are defined as the proteins with the highest intra-modular connectivity (kIN) score (top 10%) and the highest pearson correlation value with the module eigenprotein (>0.8).

#### Module correlation to DOX treatment and individual

To assess the relationship between each module’s eigenprotein and the defined trait (DOX treatment or individual), we performed a pearson correlation analysis using the cor.test function from the stats package in R.^95^ This analysis provided a correlation coefficient for each module, indicating the strength and direction of the association between the module’s expression profile and the experimental condition. The statistical significance of these correlations was determined by extracting *p* values for each module-trait correlation using the cor.test function, where *p* values less than 0.01 were determined to be significant.

#### Hub protein correlation network visualization in DOX-correlated modules

The correlation network for hub proteins in DOX-correlated modules was visualized using the igraph package in R.^96^ Abundance correlations between proteins were obtained using the get_edge_list function from BioNERO.^94^ Protein pairs with positive Pearson correlations of 0.9 or more were selected for visualization.

#### Linear modeling to identify differentially abundant proteins

We utilized the limma package to fit protein abundances to a linear model across conditions.^97^^,^^98^ We randomly selected one technical replicate per treatment group from Individual 3 so as not to confound technical and biological variation in the linear modeling process. The RUV-III corrected abundance matrix from six samples was quantile normalized using the normalizeBetweenArrays function. Drug treatment (DOX or VEH) was modeled as a fixed effect, whereas individual (IND) was treated as a random effect estimated using the duplicateCorrelation function. The linear model fitting was done using lmFit, which incorporated the block effect from the individuals and the design matrix. This model was then passed through the empirical Bayes moderation in the eBayes function to obtain moderated t-statistics. We defined contrasts in the linear model to compare the differential expression between DOX and VEH conditions such that positive log_2_-fold change values correspond to increased abundance in the DOX-treated group, and negative values correspond to decreased abundance in the DOX-treated group. The model was refitted with these contrasts, and empirical Bayes moderation was applied again to adjust the statistics. We performed multiple-testing correction with the Benjamini-Hochberg method and denoted those proteins that meet an adjusted *p*-value threshold of 0.05 as differentially abundant proteins (DAPs).

#### Comparison of the DIA and DDA datasets

We detected 4,501 proteins by DDA and removed all proteins containing at least one missing protein abundance value, resulting in 3,384 measured proteins. We detected 4,261 proteins by DIA and removed all proteins containing at least one missing protein abundance value, resulting in 3,934 measured proteins. We selected the 3,027 proteins shared between these two sets and calculated the mean log_2_ abundance for each protein across all samples. DDA and DIA protein log_2_ abundance were fit to a linear model using the lmFit function to determine the strength and significance of the correlation between datasets. We identified differentially abundant proteins in the DDA dataset as described above for DIA, using the same preprocessing and modeling parameters. We then compared the response effect sizes (log_2_ fold change) between proteins shared between the two datasets using the lmFit function to determine the strength and significance of the correlation between datasets.

#### Comparison of proteins elevated in heart tissue across modules

We sourced data on proteins whose expression is elevated in heart tissue compared to other tissue types from the Human Protein Atlas.^33^ Elevated proteins correspond to those with at least a 4-fold higher mRNA level in a particular tissue compared to any other tissue. Proteins identified as elevated in heart tissue were categorized into their respective modules within our correlation network. We then calculated their percentages relative to the total protein count within each respective module.

#### Comparison of heart ventricle tissue specificity scores across modules

Tissue specificity values for 'Heart.Ventricle' were obtained for our 4,178 proteins.^20^ We tested for differences in tissue specificity across modules using a Wilcoxon rank-sum test for each module. We compared scores between all proteins in a given module, and all proteins not contained within that module, where significant differences were denoted when *p* < 0.05.

#### Cellular localization of module proteins

We retrieved lists of proteins classified as signal peptides, voltage-gated channels, secreted, intracellular, membrane-bound, or plasma proteins from the Human Protein Atlas database.^33^ We utilized the UniProt database^34^ to identify proteins experimentally shown to be located in subcellular structures including autophagosomes, membranes, cytoplasm, cytoskeleton, endoplasmic reticulum, Golgi apparatus, lysosomes, mitochondria, nucleus, and sarcomere. We classified each protein in the correlation network according to the above annotations, and performed a Fisher’s exact test to determine which modules were enriched for proteins from a particular classification. Fisher’s exact test *p* < 0.05 was considered significant.

#### Biological process enrichment across modules

To interpret the biological significance of the WGCNA-derived modules correlated with the DOX treatment, enrichment analysis was performed based on annotated Gene Ontologies (GO).^99^ The enrichGO function from the clusterProfiler R package was used to test the enrichment of terms associated with biological processes against the background of all network proteins.^100^ Enriched terms were those with a Benjamini-Hochberg adjusted *p* < 0.05.

#### Functional categorization of module proteins

We retrieved lists of proteins classified as human transcription factors,^35^ RNA binding proteins (RBPs),^37^ enzymes and transporter proteins,^33^ and mammalian stress granule (MSG) proteins.^36^ Enrichment of different protein categories within each module was determined by Fisher’s exact test. Enriched terms were those with a Benjamini-Hochberg adjusted *p* < 0.05.

#### Protein family enrichment amongst module proteins

We queried UniProt for protein family names for the set of network proteins.^34^ We assigned each protein to its set of families and performed a Fisher’s exact test to determine which modules were enriched for proteins from a particular family. Those with a Benjamini-Hochberg adjusted *p* < 0.05 were considered significant.

#### pQTL protein enrichment

We obtained plasma pQTL data from the UK Biobank.^17^ We selected pQTLs that were identified independent of genetic ancestry. pQTLs were further classified as *cis*-pQTLs or *trans*-pQTLs. For pQTL protein enrichment analysis, we collated the set of unique pQTL proteins. We tested for enrichment of pQTL proteins among hub proteins, DOX-correlated proteins, and DOX-correlated hub proteins, compared to the set of proteins not contained within each of those sets using the Fisher’s exact test. A Fisher’s exact *p* < 0.05 was considered statistically significant.

#### Comparison of pQTL SNP effect sizes

From the aforementioned set of *cis*-pQTLs and *trans*-pQTLs, we selected the SNP with the highest effect size for each protein. We tested for differences in effect sizes between DOX-correlated and non-DOX-correlated proteins, hub proteins and non-hub proteins, and DOX-correlated hub proteins and non-DOX-correlated hub proteins using a Wilcoxon rank-sum test, where a *p* < 0.05 was considered statistically significant.

#### Proportion of pQTLs across network protein connectivity deciles

For each protein in the network, we calculated the normalized intra-modular connectivity by taking the kIN of each protein and dividing it by one less than the number of module connections. Proteins were assigned to one of two groups based on their DOX-correlation status, and stratified into deciles based on their normalized intra-modular connectivity. We then calculated the proportion of proteins in each decile that were pQTLs (either *cis-* or *trans*-pQTLs).

#### pLI, pHaplo and pTriplo comparisons across modules

We obtained probability of loss-of-function intolerance (pLI) scores^101^ and probability of Haploinsufficiency (pHaplo) and Triplosensitivity (pTriplo) scores.^44^ We tested whether the set of scores for each metric was different for each module compared to all proteins in the network outside the module using the Wilcoxon rank-sum test. *p* < 0.05 was considered to be significant.

#### Comparisons between pLI scores and network connectivity

Proteins with a pLI ≥0.9 are considered intolerant to mutation, while proteins with a pLI ≤0.1 are considered tolerant to mutation. We compared the normalized kIN values of mutation-intolerant proteins to mutation-tolerant proteins using the Wilcoxon rank-sum test. *p* < 0.05 was considered to be significant.

We used three types of connectivity metrics to describe how each protein in the network related to pLI scores across connectivity deciles. Total connectivity was calculated by summing the weighted correlations between each protein and all other proteins in the network (kTotal). Normalized kIN was used as described above. Extra-modular connectivity (kOut), the correlation between proteins within each module to all proteins outside the module, was normalized by dividing kOut by the number proteins outside the target protein’s module. Proteins were assigned into two groups based on their DOX-correlation status, and deciles generated for each aforementioned type of connectivity.

To ascertain enrichment for mutation-tolerant or intolerant proteins among hub proteins, DOX-correlated proteins, and DOX-correlated hub proteins, the Fisher’s exact test was conducted. A Fisher’s exact *p* < 0.05 was considered statistically significant.

#### CVD GWAS enrichment testing

We obtained GWAS summary statistics from the GWAS catalog.^50^ We downloaded summary statistics for “heart disease” (EFO_0003777) to obtain risk proteins for “atrial fibrillation” and “heart failure”. As per the catalog’s metadata, each trait is associated with a set of SNPs and each SNP is associated with a ‘mapped gene’. If the SNP is located in a chromosomal region that falls within a gene, then that gene is considered the mapped gene. For intergenic SNPs, SNPs are mapped to the nearest gene upstream or downstream of its chromosomal location. These 333 mapped AF genes and 115 mapped HF genes were intersected with our set of iPSC-CM expressed proteins yielding 70 AF and 20 HF proteins to interrogate for enrichment across modules. Module-wise enrichment testing was then performed by comparing to the set of all proteins not contained within the module of interest. This analysis involved constructing contingency tables for each module-gene set pair and conducting Fisher’s exact tests to determine the overlap.

#### CVD risk protein interactor enrichment testing

Using the set of ‘heart disease’ CVD risk proteins as mentioned above, protein UniProt IDs were uploaded to STRINGdb and PPI networks were generated.^102^ We focused on proteins with experimental interaction evidence (confidence score ≥0.9). The protein interactors for CVD risk proteins were denoted “CVD protein interactors”. To ascertain enrichment for CVD proteins and CVD protein interactors among hub proteins, DOX-correlated proteins, and DOX-correlated hub proteins, the Fisher’s exact test was conducted. A Fisher’s exact *p* < 0.05 was considered statistically significant.

#### pLI comparison between CVD proteins and CVD protein interactors

Using the set of CVD proteins and CVD protein interactors as mentioned above, we assigned each protein their pLI score as previously described. We compared the pLI values between the two groups using the Wilcoxon rank-sum test. *p* < 0.05 was considered to be significant.

We also generated a degree-randomized PPI network with resampling to ensure robustness of results. We utilized the set of CVD proteins and their physical protein-protein interaction partners, as determined by STRINGdb with confidence scores above 0.9. The degree of each GWAS protein within the physical PPI network was calculated, resulting in a degree distribution for the CVD proteins. This distribution highlighted the percentage of proteins with varying degrees of interaction. CVD proteins were assigned group names based on their degree, and we subsequently calculated the degree for every protein in the entire network. Proteins in the network with degrees similar to those of the CVD proteins were assigned to the same group, while those with degrees not captured by the CVD-proteins were assigned to the group with the nearest degree. Each protein in the network was thus assigned to a group reflective of the degree distribution of the GWAS proteins, ensuring comprehensive sampling without excluding proteins based on their degree. We resampled while maintaining a degree-based proportion comparable to that of the original CVD proteins. We simulated 10,000 subnetworks among sampled proteins and their interactors, comparing pLI scores between CVD proteins and their physical interactors using the Wilcoxon rank-sum test.

#### PPI network construction

UniProt IDs for all proteins in the iPSC-CM network were imported into Cytoscape^103^ to generate a PPI network. PPIs were determined using the STRINGdb application in Cytoscape. We selected the ‘physical subnetwork’ of PPIs with confidence scores >0.4, prior to later sub-setting PPIs with confidence scores >0.9 in R. The combination of both moderate and stringent cutoffs was used for sensitivity analysis of our results. We then annotated proteins in the network by whether they were DOX-correlated, hub proteins or CVD proteins, as well as by which module each protein belonged to. Edges in the network represent the WGCNA-derived weighted correlation between proteins. We next generated a subnetwork from the total network by selecting PPIs where at least one protein in the PPI was a CVD protein to center our analyses on the differences between CVD proteins and their direct physical protein interactors. We then used the tidygraph^104^ and ggraph^105^ packages in the R programming environment to visualize a network of CVD proteins and their physical protein interactors.

### Quantification and statistical analysis

All statistical analyses were performed using R version 4.4.1 unless otherwise noted. Analyses were performed using R packages including: WGCNA, limma, edgeR, clusterProfiler, ggplot2, igraph, AnnotationDbi, biomaRt, pheatmap, BioNERO, dynamicTreeCut, ggraph, ruv, impute, org.Hs.e.g.,.db, stats, SummarizedExperiment, and tidygraph (see key resources table). Statistical details—including test types, *n* values, *p* values and adjusted *p* values are provided in the figure legends, results and STAR Methods sections, and Supplemental Tables.

For γH2AX immunofluorescence analysis (Figure 1C), n represents three biological replicates derived from three independent iPSC-CM lines (one per individual). For proteomics-based comparisons, *n* = 10 total samples, derived from iPSC-CMs generated from three individuals. Individuals 1 and 2 each contributed one sample per treatment group (DOX and VEH), while Individual 3 contributed three technical replicates per treatment group. For network analyses and enrichment testing, n represents the number of proteins in each module or test set, as detailed in Table S2.

A two-tailed paired Student’s t test was used to compare γH2AX-positive nuclei percentages between DOX- and VEH-treated cardiomyocytes (*p* < 0.05). Pearson correlation (via cor.test, R stats package) was used to assess relationships between module eigenproteins and traits (e.g., DOX treatment, individual), with *p* < 0.01 considered significant. Differential abundance analysis of proteins was performed using a linear model, and multiple testing correction was applied using the Benjamini-Hochberg method (adjusted *p* < 0.05). The Wilcoxon rank-sum test was used to compare distributions of tissue specificity scores across modules, SNP effect sizes in pQTL proteins, mutation tolerance metrics (pLI, pHaplo, pTriplo) across modules, and connectivity distributions of mutation-tolerant vs. intolerant proteins. Fisher’s exact test was applied to assess enrichment of Gene Ontology (GO) terms and biological processes, protein family domains and subcellular localization categories, pQTL overlap and mutation intolerance across hub proteins, GWAS gene/protein enrichment in modules or networks, and drug target enrichment in DOX-correlated modules.

Unless otherwise specified, *p* < 0.05 was considered statistically significant. Multiple testing was performed using Benjamini-Hochberg false discovery rate (FDR) control where applicable. Values are expressed as mean unless otherwise indicated. Error bars represent mean ± standard deviation (SD) as noted in Figure 1C.

### Additional resources

This study does not involve a clinical trial and is not registered in a clinical trial database.

## Supplemental information

Document S1. Figures S1–S16

Table S1. iPSC-CM sample attributes, related to Figure 1

Table S2. Detected proteins, their module assignment (gray = module unassigned), output of differential abundance analysis (logFC >0 up in DOX), and designation as various features (0 = False, 1 = True), related to Figures 2,3,4,6

Table S3. Significantly enriched biological processes (BP), molecular functions (MF), and cellular components (CC) across modules (adj. *p* value <0.05), related to Figure 2

Table S4. GWAS-defined CVD proteins expressed in the iPSC-CM network and their physical protein interactors (STRINGdb confidence score >0.9), related to Figure 6

Data S1. Raw protein abundance matrix, related to Figure 1

Data S2. WGCNA-derived weighted correlations of protein abundance across all pairs of proteins in the iPSC-CM DOX response network, related to Figure 2
