# Supplementary material for: DNA-damage-associated protein co-expression network in cardiomyocytes informs on tolerance to genetic variation and disease
Source: iScience. 2025 Apr 18;28(5):112474. doi: 10.1016/j.isci.2025.112474 (PMC12135479; doi:10.1016/j.isci.2025.112474)
Supplement: Document S1. Figures S1–S16 [file mmc1.pdf]

iScience, Volume *28*

Supplemental information

DNA-damage-associated protein co-expression network in cardiomyocytes informs on tolerance to genetic variation and disease

Omar Darrel Johnson, Sayan Paul, José Angel Gutiérrez, William Kent Russell, and Michelle Claire Ward

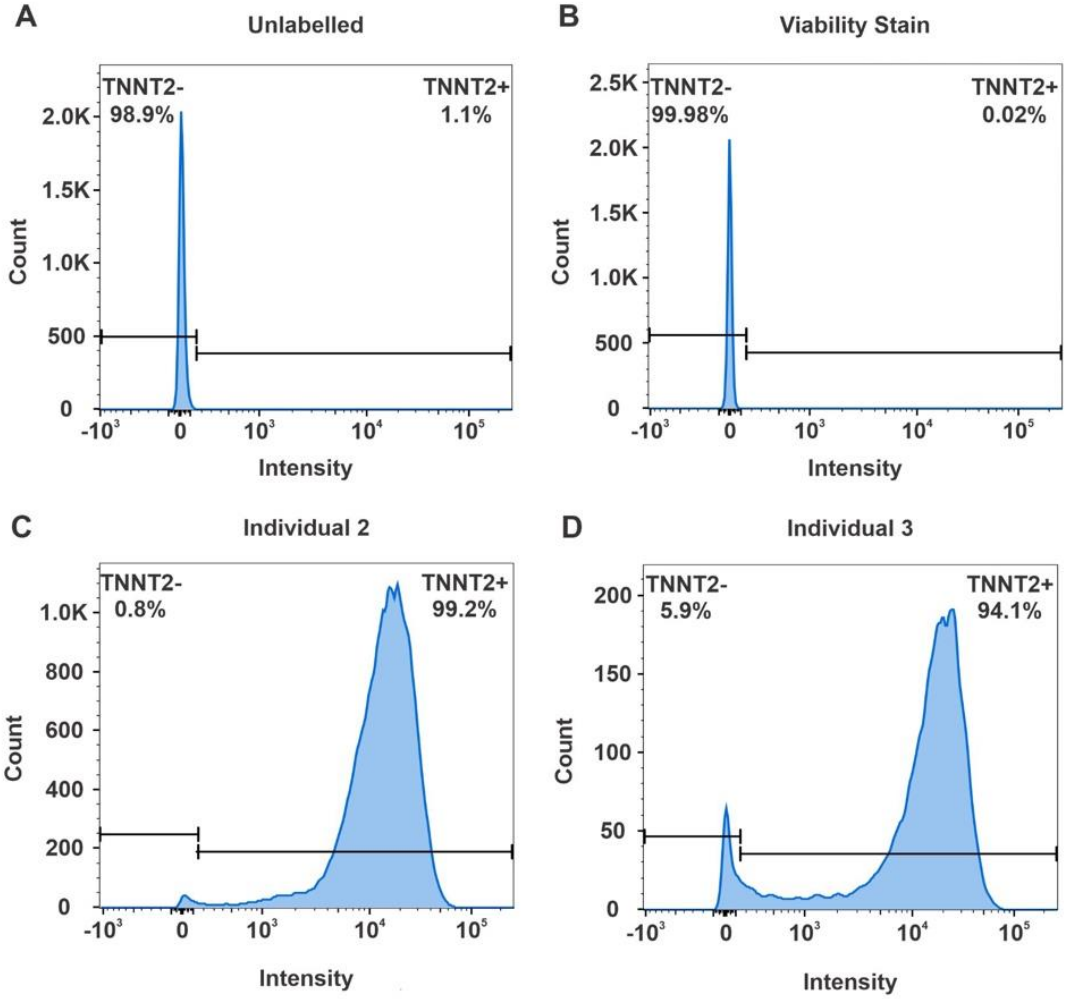

Figure S1: iPSC-derived cardiomyocytes can be generated at high purity, related to Figure 1. (A) Proportion of troponin (TNNT2) positive cells in unlabeled iPSC-CMs determined by flow cytometry. **(B)** Proportion of TNNT2-positive cells in iPSC-CMs labeled with viability stain only. **(C)** Proportion of live TNNT2-positive cells in Individual 2 iPSC-CMs. **(D)** Proportion of live TNNT2-positive cells in Individual 3 iPSC-CMs.

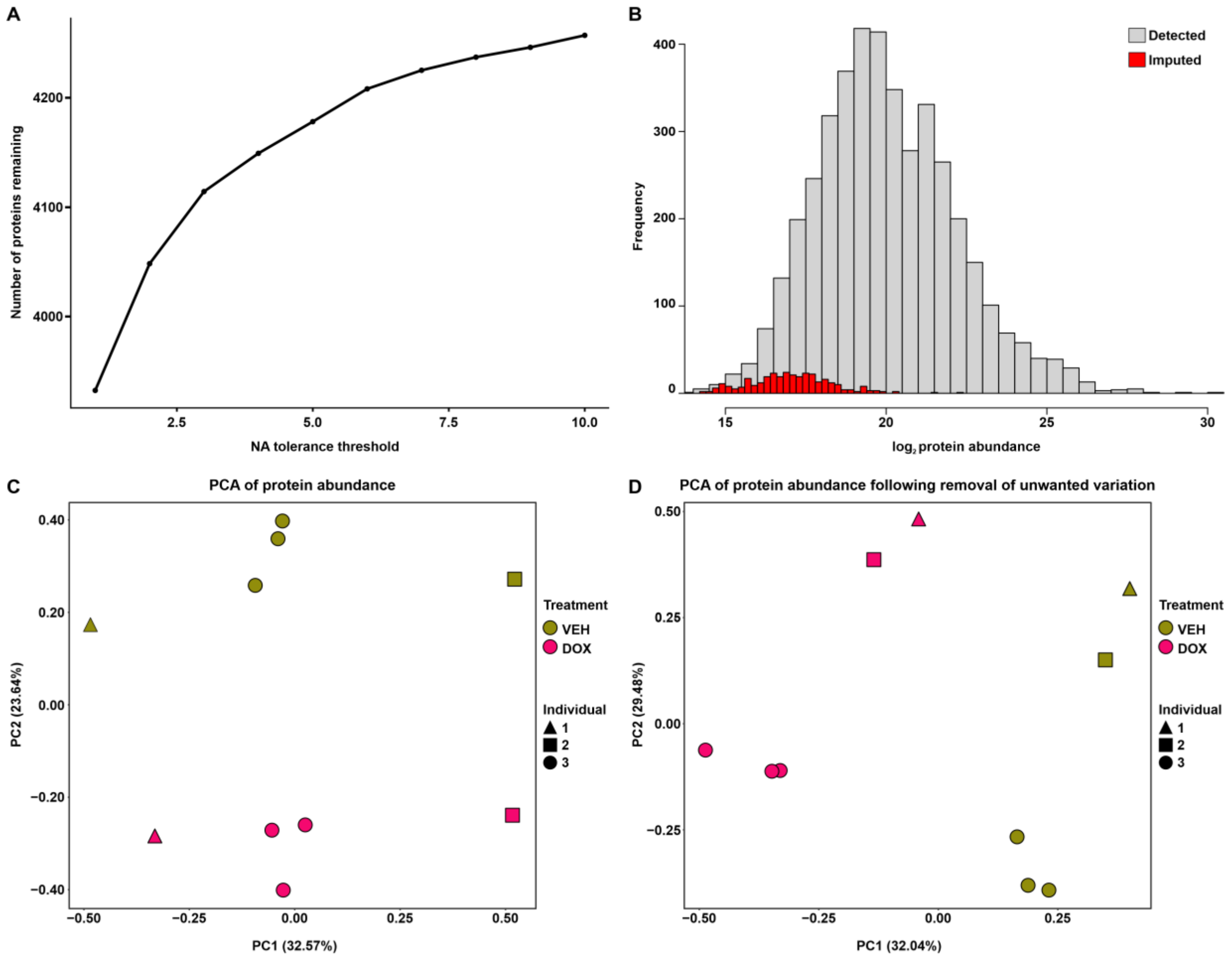

Figure S2: DOX treatment is the primary contributor to variation in protein abundance following pre-processing, related to Figure 1. (A) The number of measured proteins at different sample thresholds of missing values. **(B)** Distribution of detected (grey) and imputed (red) $\log_2$ protein abundance values. **(C)** Principal component analysis (PCA) of $\log_2$ protein abundance data. Samples are colored by treatment (DOX: pink, VEH: olive) and shaped by individual (Individual 1: triangle, Individual 2: square, Individual 3: circle). **(D)** PCA of $\log_2$ protein abundance values after the removal of unwanted technical variation.

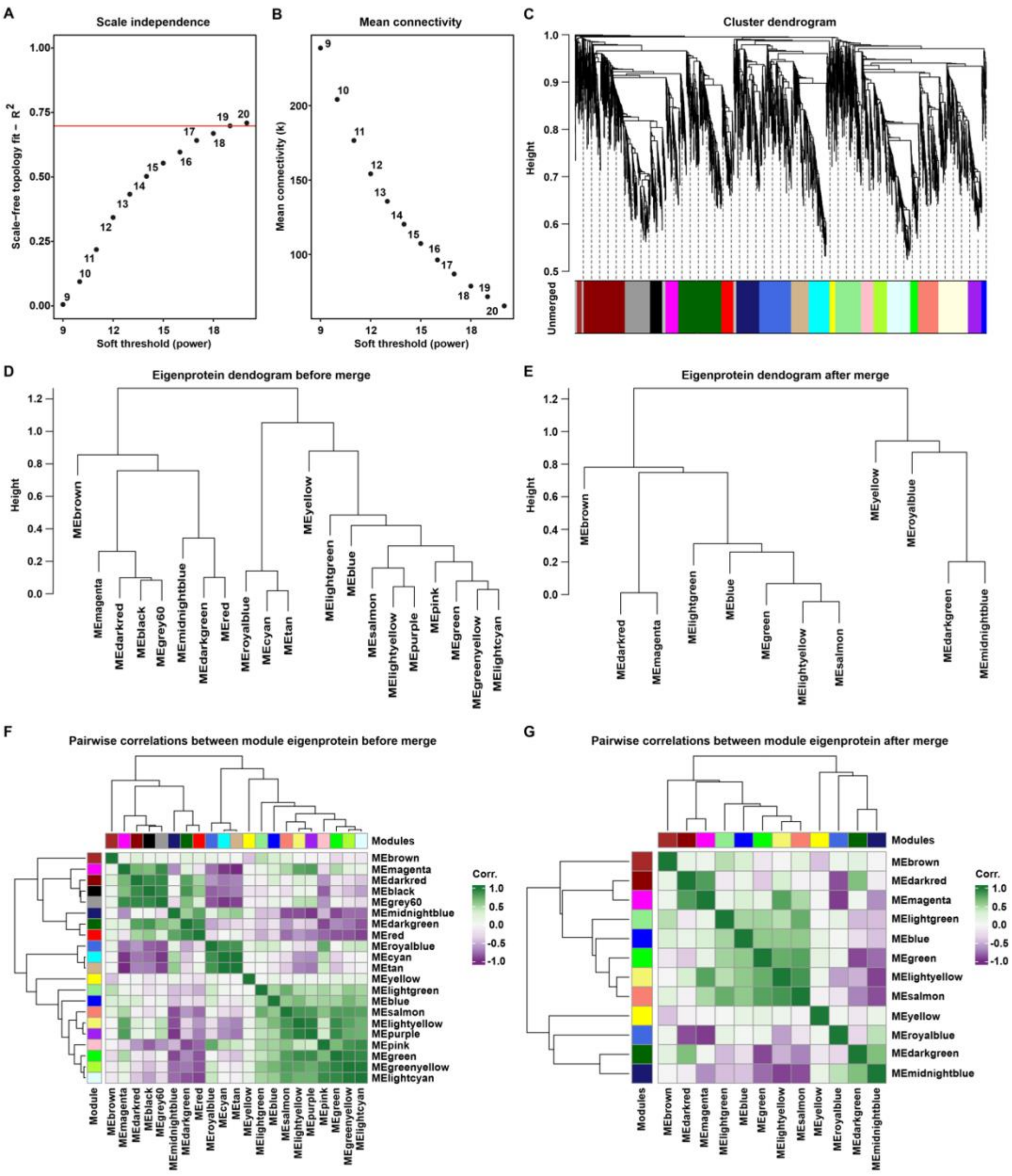

Figure S3: Weighted protein co-expression network with scale-free topology generates co-expressed modules that can be summarized by eigenproteins, related to Figure 2. (A) Network fit to a scale-free topology across soft power thresholds. Fit is determined by the log-log correlation between the connectivity probability P(k) and connectivity (k). The red line indicates the threshold selected (20). **(B)** Mean network connectivity (k) across soft power thresholds. **(C)** Cluster dendrogram of the network, where height represents the dissimilarity of clusters across modules. Each module is shown by a different color. **(D)** Simplified dendrogram from (C) containing 21 co-expressed modules, where each module is represented by its eigenprotein (ME). **(E)** Eigenprotein dendrogram after merging similar modules with a pearson correlation > 0.85, yielding 12 co-expressed modules. **(F)** Correlation heatmap of module eigenproteins shown in (D) of the unmerged network. **(G)** Correlation heatmap of module eigenproteins shown in (E) of the network after merging modules.

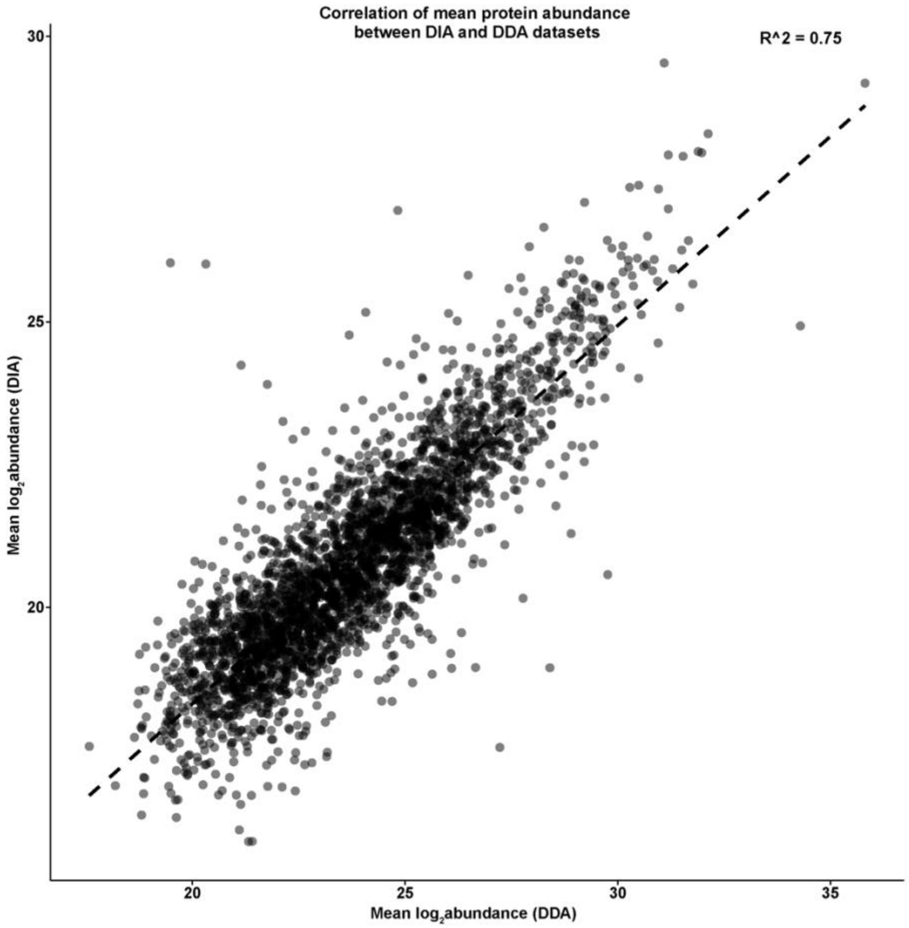

Figure S4: Protein detection and abundance is similar across DIA and DDA protein acquisition methods, related to Figure 2. Correlation of mean $\log_2$ protein abundance of the 3,027 proteins present in all samples across Data-Dependent Acquisition (DDA) and Data-Independent Acquisition (DIA) datasets. The dashed line represents the line of best fit. Correlation $P < 0.001$.

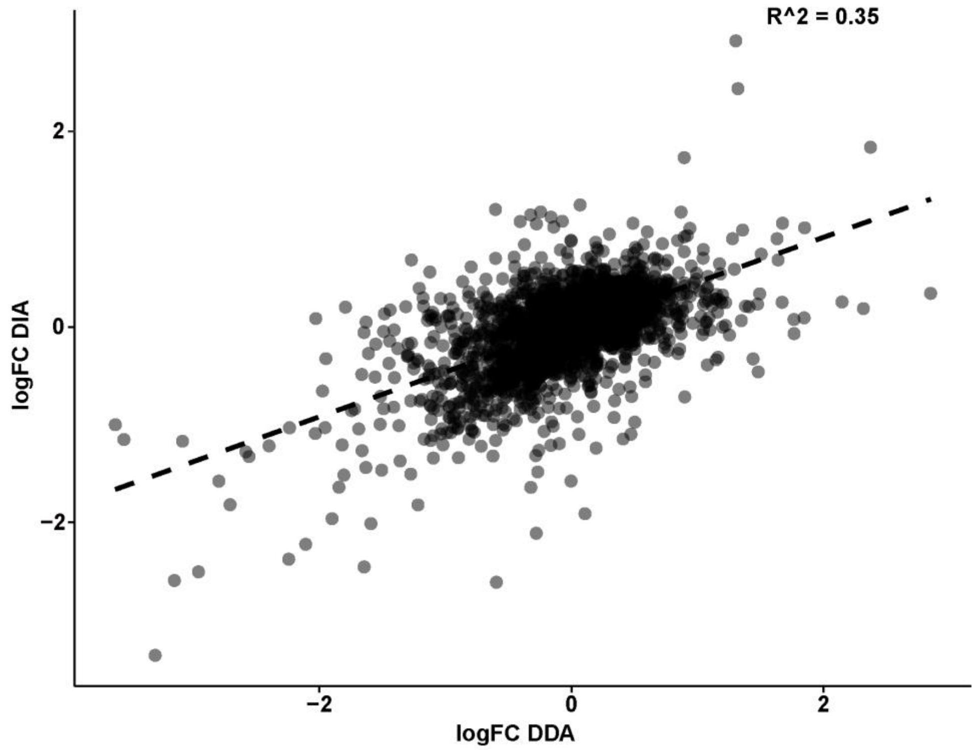

Figure S5: Response to DOX between DDA and DIA protein acquisition methods is correlated, related to Figure 2. Log_2 fold change between DOX and VEH for proteins detected and imputed using the Data-Dependent Acquisition (DDA) and Data-Independent Acquisition (DIA) methods is shown. The dashed line indicates the best fit line. Correlation $P < 0.001$.

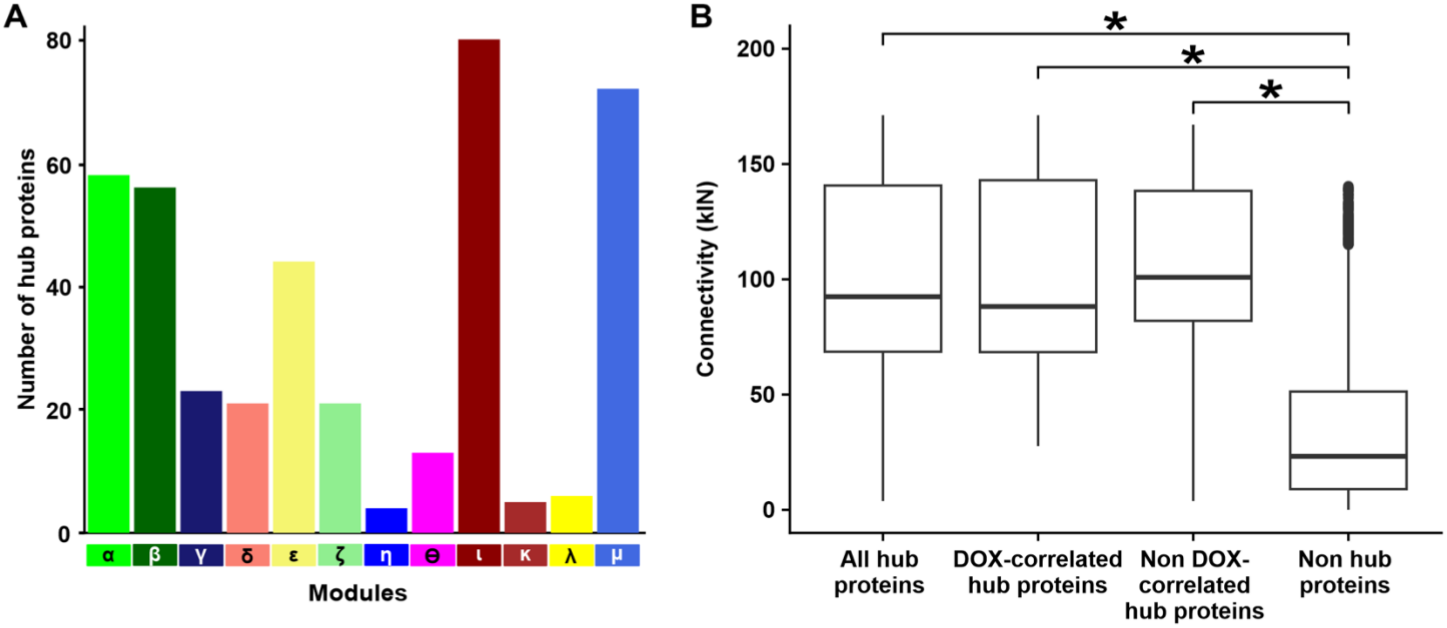

Figure S6: Hub proteins are represented across all co-expressed modules and have greater intra-modular connectivity than non-hub proteins irrespective of DOX-correlation status, related to Figure 2. (A) Modules are ordered from α, the module with the strongest correlation to DOX, to μ, the module with the weakest correlation to DOX. **(B)** Distribution of connectivity scores (kIN) for four categories of proteins: All hub proteins (n = 403), DOX-correlated hub proteins (n = 202), non-DOX-correlated hub proteins (n = 201), and network proteins that are non-hub proteins (n = 3,775). Asterisk denotes a statistically significant difference in kIN between conditions ($P < 0.05$).

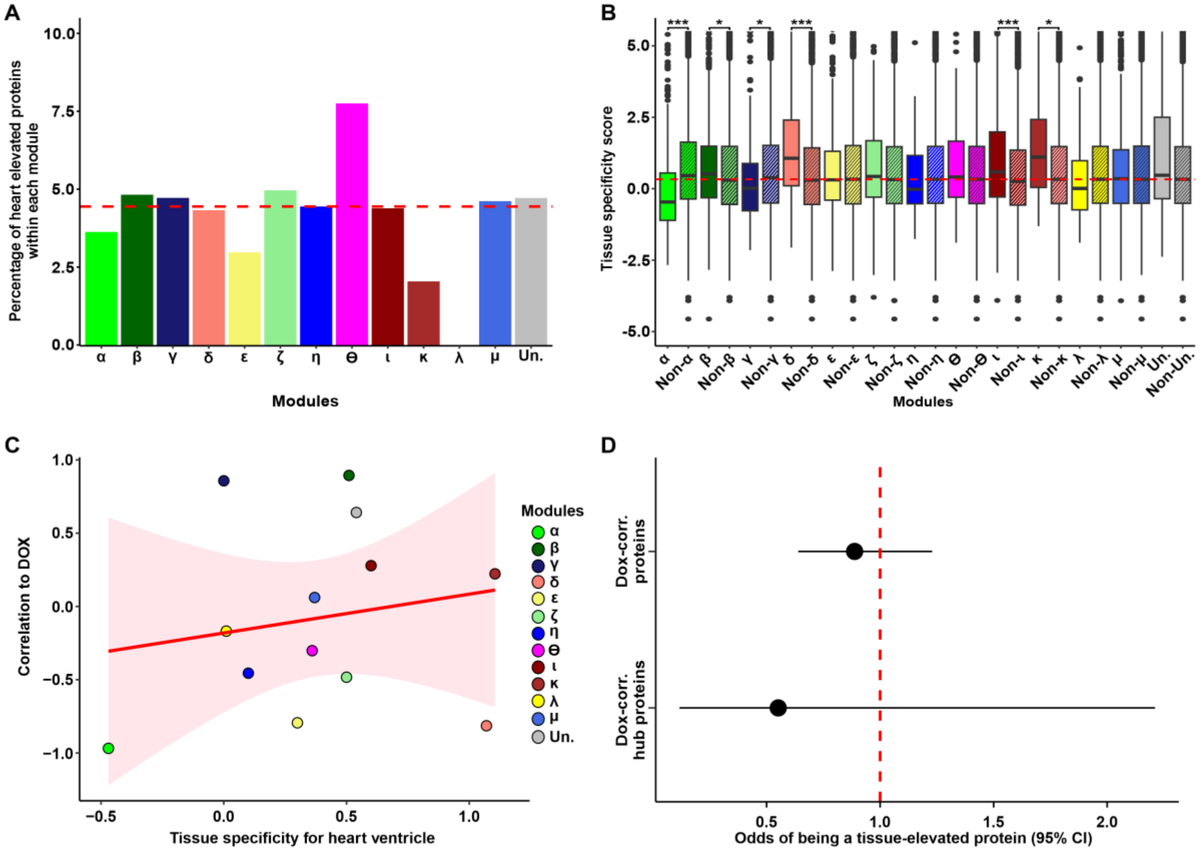

Figure S7: Modules across the network show heterogeneity for tissue specificity, related to Figure 2. (A) Percentage of each module in the network that consists of proteins annotated as having increased expression in heart relative to other tissues [S1]. Dashed red line represents the median percentage of heart elevated proteins across all modules. **(B)** Comparison of heart ventricle tissue specificity scores for each module (x) in the network compared to proteins in all other modules (Non-x)[S2]. The red dashed line indicates the median tissue specificity score for the network. Asterisk represents statistically significant differences in tissue-specificity scores between module proteins and non-module proteins (*$P < 0.01$, ***$P < 0.0001$). **(C)** Median tissue specificity scores for each module and the correlation of each module to DOX. The red line represents the best-fit regression line, with the pink-shaded area showing the 95% confidence interval for the estimated relationship between the variables (rho = 0.18). **(D)** Enrichment of tissue-elevated proteins amongst DOX-correlated proteins and DOX-correlated hub proteins.

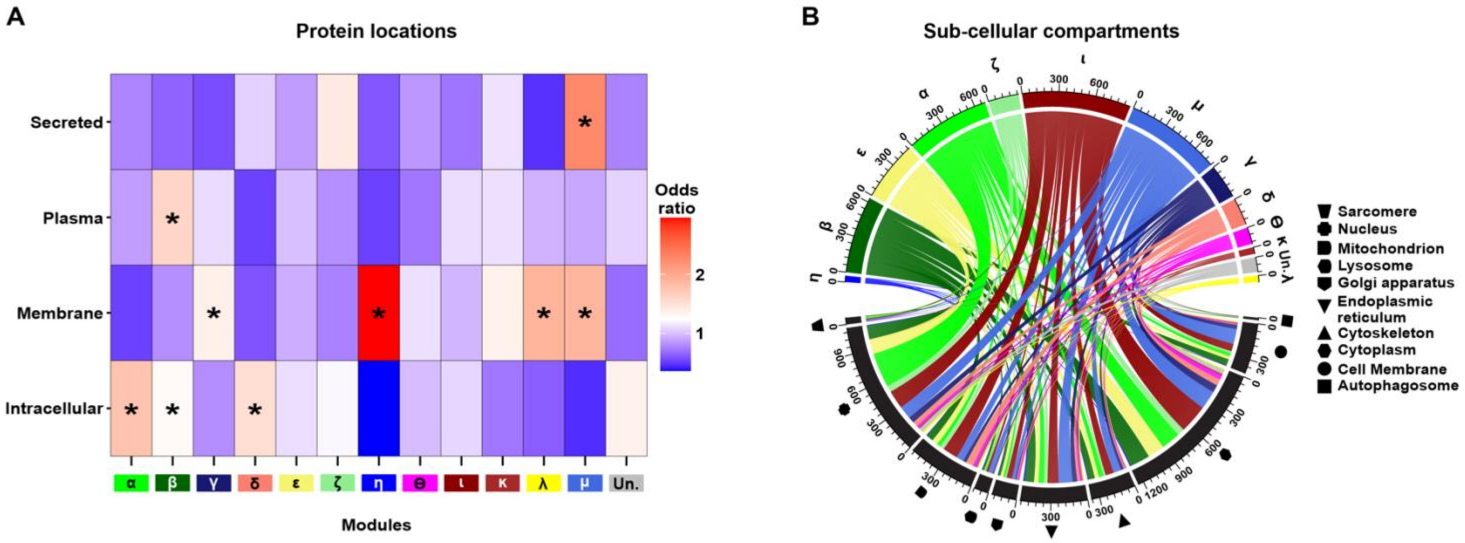

Figure S8: Network modules display heterogeneity for cellular localization, related to Figure 2. (A) Enrichment of module proteins across cellular and extracellular compartments. Asterisk represents locations with a significant enrichment of module proteins ($P < 0.05$). **(B)** Distribution of module proteins across subcellular compartments. Colors represent the set of proteins in each module and shapes represent the cellular compartments. Numbers indicate the number of proteins in each module or compartment.

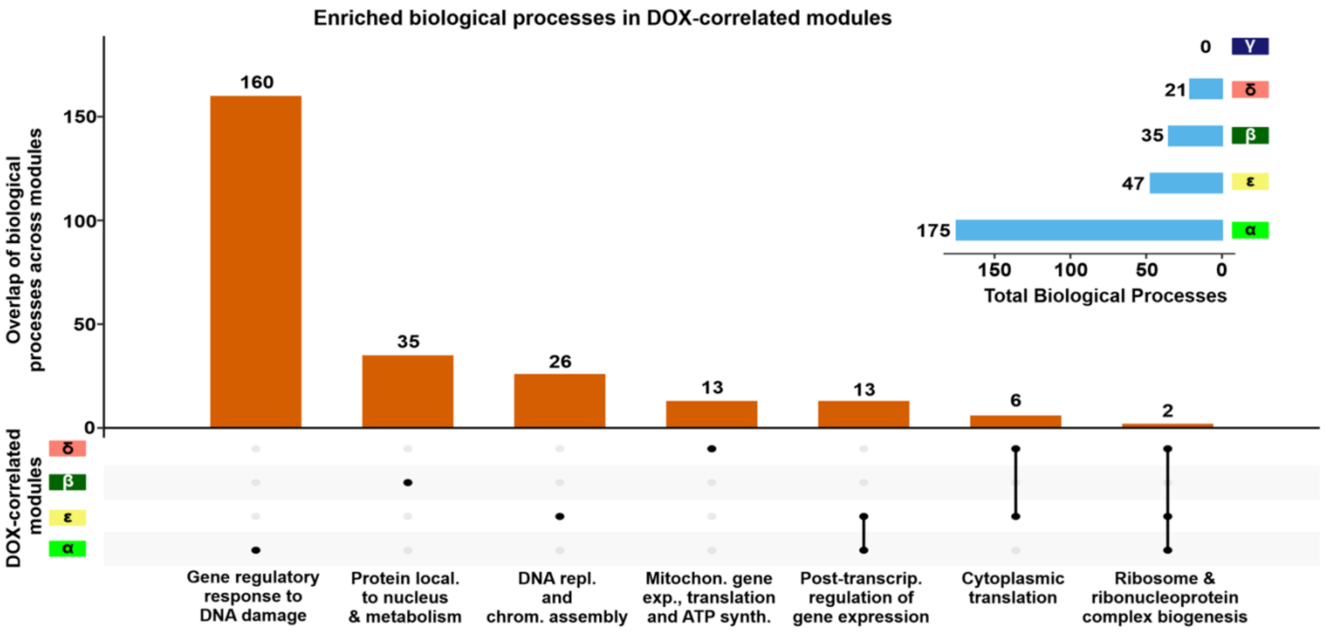

Figure S9: DOX-correlated module proteins are enriched for distinct biological processes, related to Figure 2. The total number of enriched biological processes (adjusted $P < 0.05$) across DOX-correlated modules and their overlap between modules. A curated set of terms representing the top enriched processes is indicated.

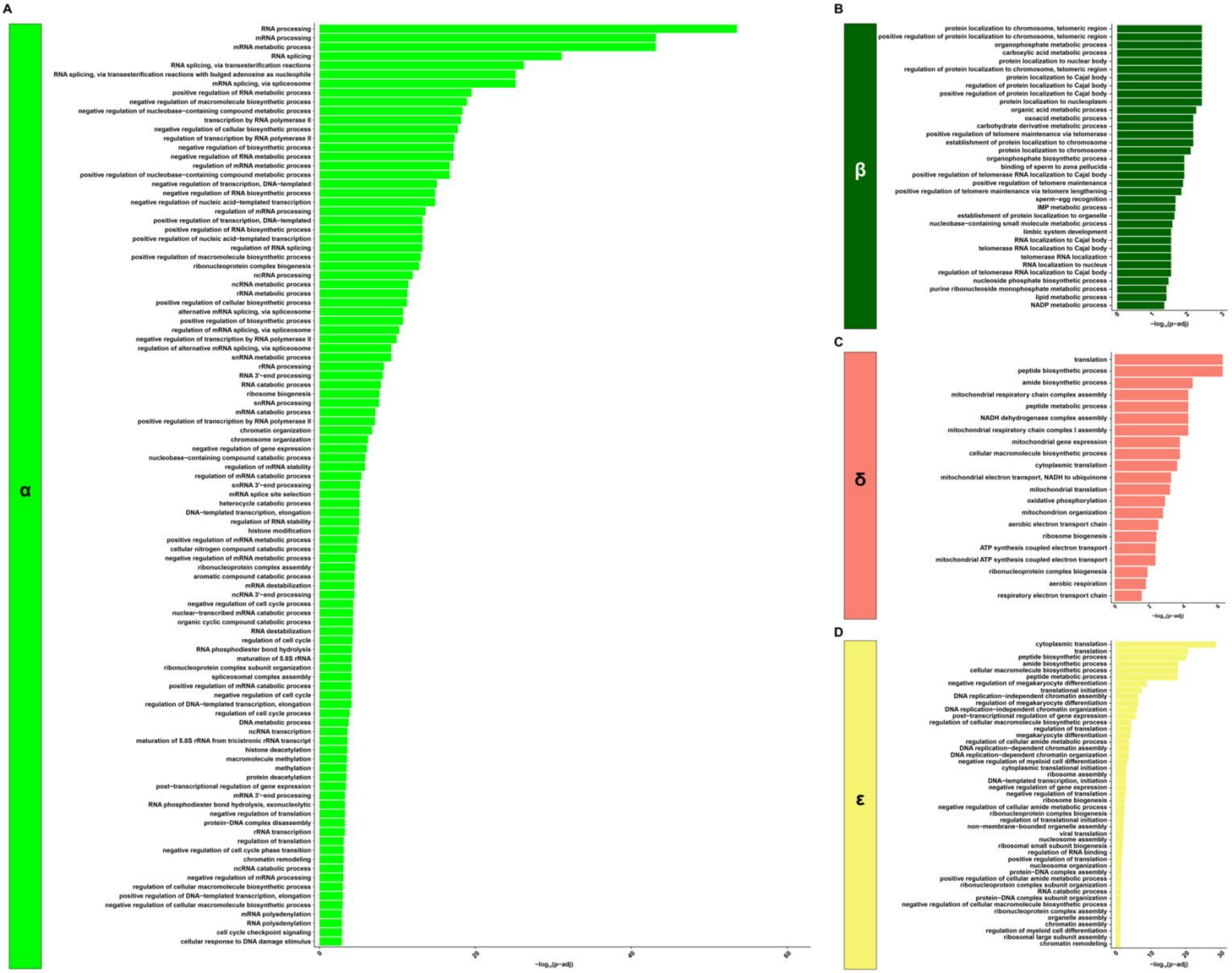

Figure S10: DOX-correlated modules are enriched for many biological processes, related to Figure 2. (A) Biological processes enriched in the α module (See Table S3 for complete term list). **(B)** Biological processes enriched in the β module. **(C)** Biological processes enriched in the δ module. **(D)** Biological processes enriched in the ε module. Enriched processes are defined by Fisher's exact test adjusted $P < 0.05$.

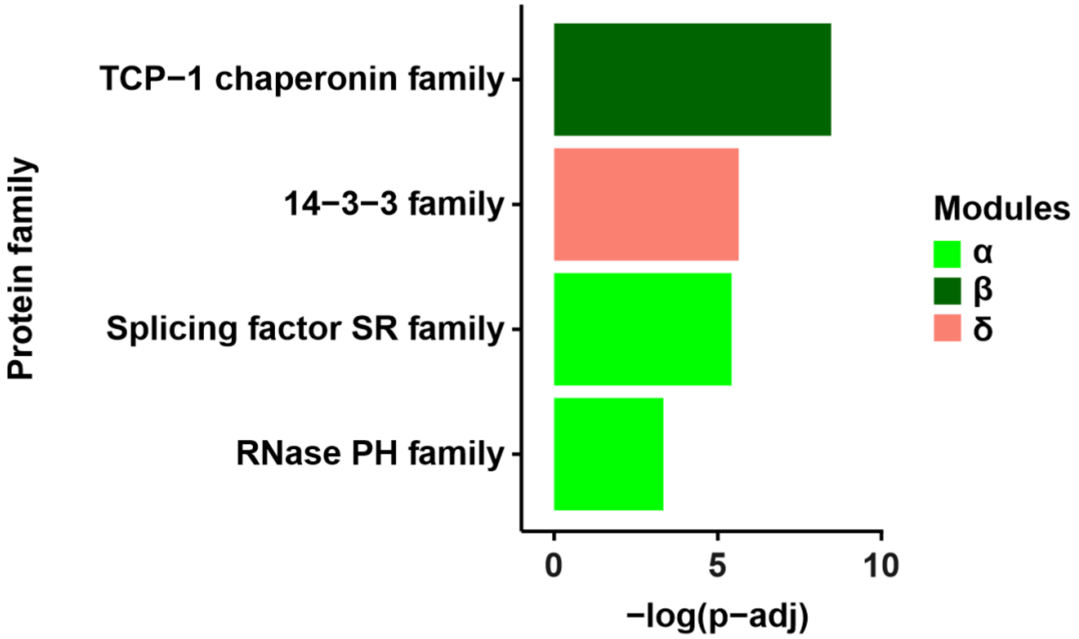

Figure S11: DOX-correlated modules differ by their enriched protein families, related to Figure 2. DOX-correlated modules α, β, and δ are ordered by their enrichment for protein families. Enrichment is determined by Fisher's exact test adjusted $P < 0.05$.

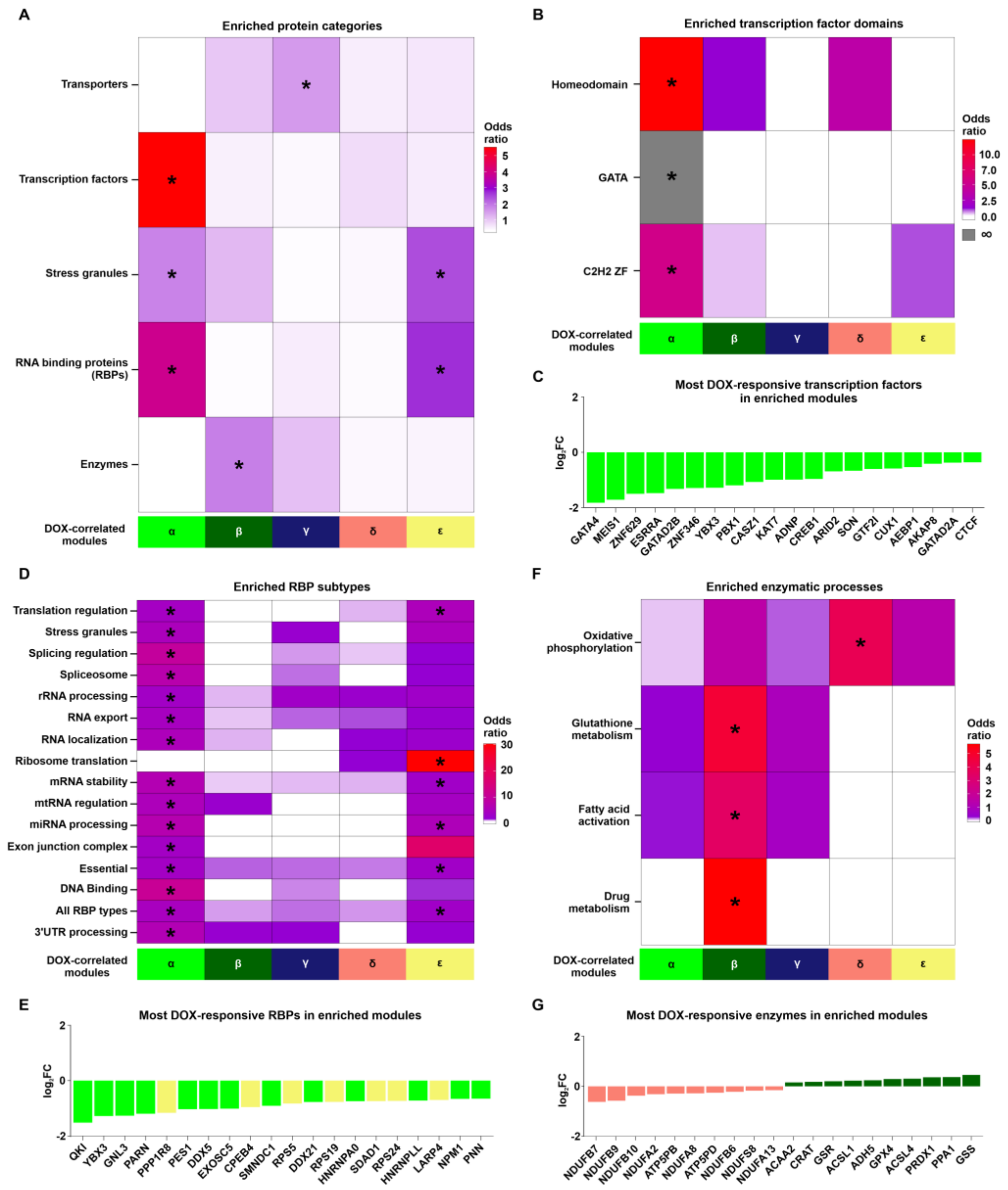

Figure S12: DOX-correlated modules are enriched for proteins involved in gene expression, post transcriptional regulation and enzymatic function, related to Figure 2. **(A)** Enrichment of proteins of different functional categories amongst DOX-correlated module proteins. Asterisk represents protein categories with a significant enrichment of module proteins (*adjusted $P < 0.05$). **(B)** Enrichment of proteins annotated by transcription factor binding domains amongst DOX-correlated module proteins. Grey shading indicates an infinite likelihood due to all transcription factor proteins with the particular binding domain being in only one module. **(C)** Top 20 most DOX-responsive transcription factors across DOX-correlated modules represented by $\log_2$ fold change from the pairwise differential abundance test. **(D)** Enrichment of RNA binding protein (RBP) types amongst DOX-correlated module proteins. **(E)** Top 20 most DOX-responsive RBPs across DOX-correlated modules. **(F)** Enrichment of proteins annotated by enzymatic process amongst DOX-correlated module proteins. **(G)** Top 20 most DOX-responsive enzymes across DOX-correlated modules.

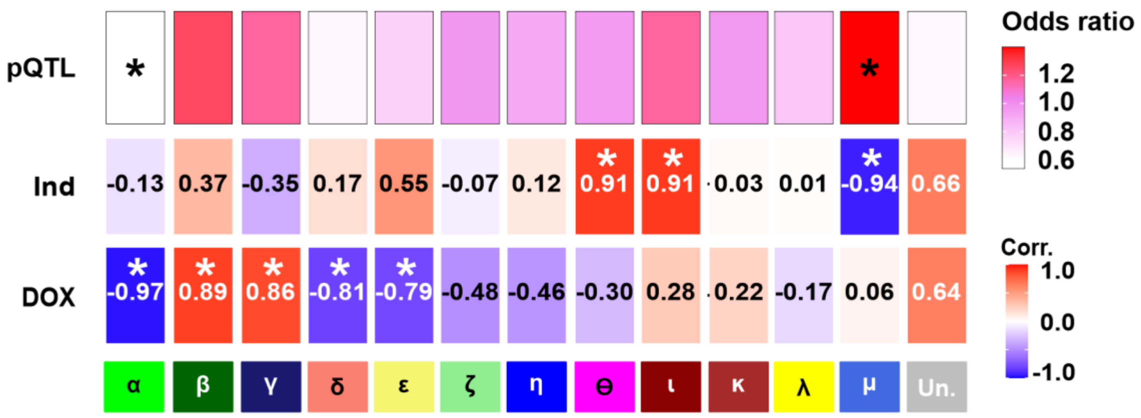

Figure S13: The only module enriched for pQTLs is correlated to individual and not to DOX, related to Figure 2. Enrichment of pQTLs (OR > 1; *adjusted $P < 0.05$) amongst module proteins in the network. The α module is significantly depleted for pQTLs (OR < 1; *adjusted $P < 0.05$). The module correlation to DOX and Individual (Ind) from Figure 1 is included for reference.

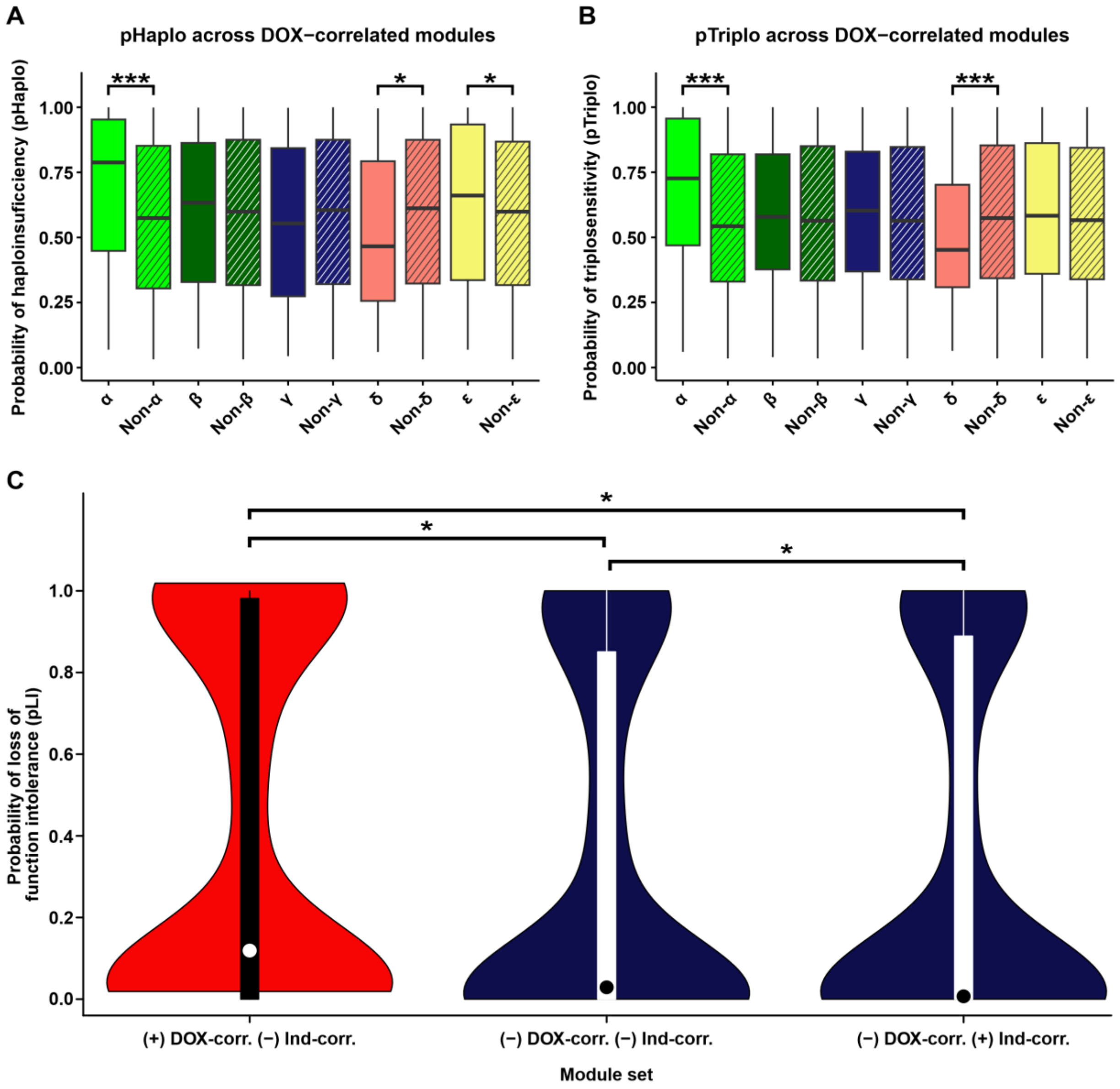

Figure S14: DOX-correlated proteins are sensitive to gene dose and have greater pLI than non-DOX-correlated modules, related to Figure 4. (A) Probability of haploinsufficiency (pHaplo) for all proteins in each DOX-correlated module (x), and all proteins outside of the module (Non-x). Asterisk represents a significant difference in scores between module-specific proteins and all proteins outside of modules (*$P < 0.01$, ***$P < 0.0001$). **(B)** Probability of triplosensitivity (pTriplo) for all proteins in each DOX-correlated module (x), and all proteins outside of the module (Non-x). **(C)** pLI amongst DOX-correlated modules (red, left), Non-DOX-correlated modules that are not correlated to Individual (Ind; blue, middle), and Non-DOX-correlated modules that are correlated to Ind (blue, right). Asterisk represents a significant difference between module sets (*$P < 0.05$).

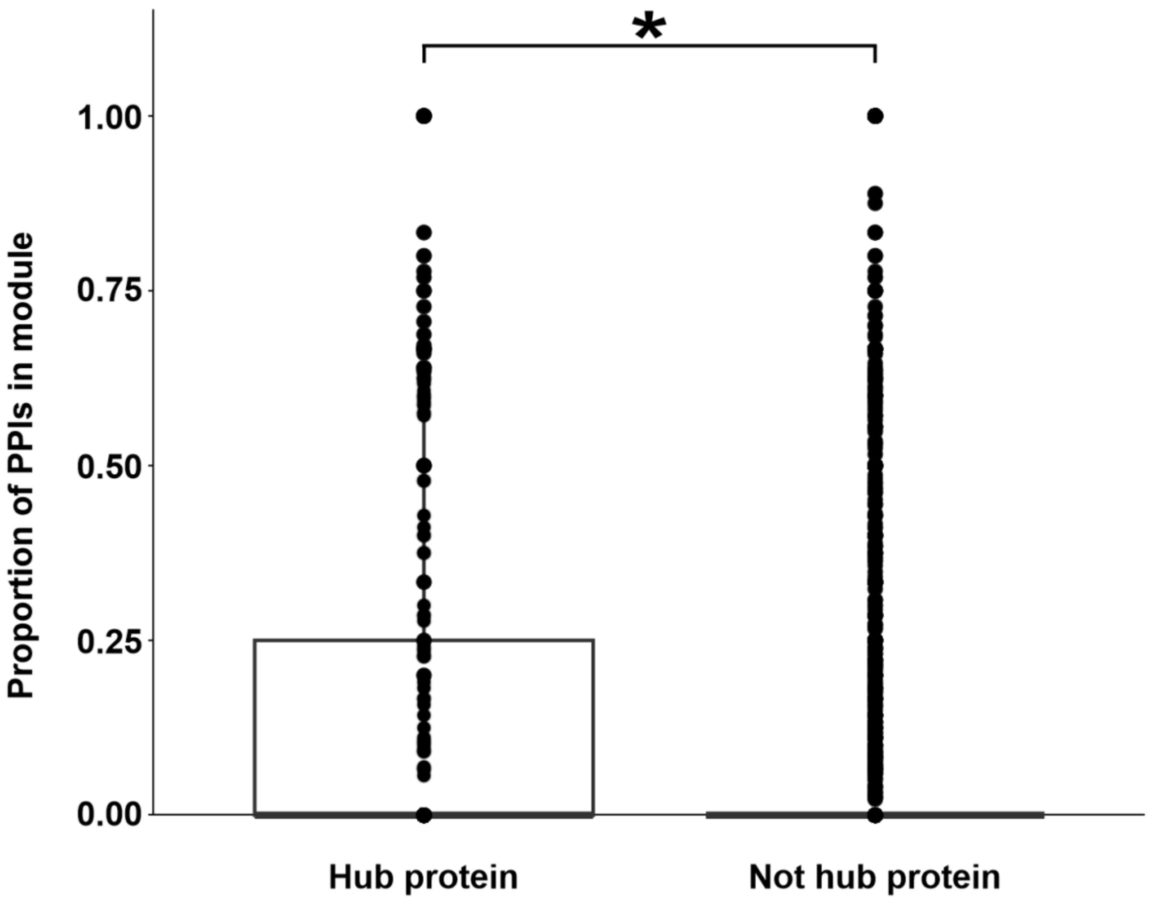

Figure S15: Hub proteins are more likely to be co-expressed with proteins they physically interact with than proteins that are not hubs, related to Figure 6. Proportion of protein-protein interactions (PPIs) where both interactors are contained within the same co-expression module for hub proteins and non-hub proteins. PPIs of expressed proteins were obtained from STRINGdb [S3], where a confidence score of ≥ 0.9 is used as the threshold for interaction. Asterisk denotes a statistically significant difference in the proportion of PPIs within the same module between hub and non-hub proteins ($P < 0.05$).

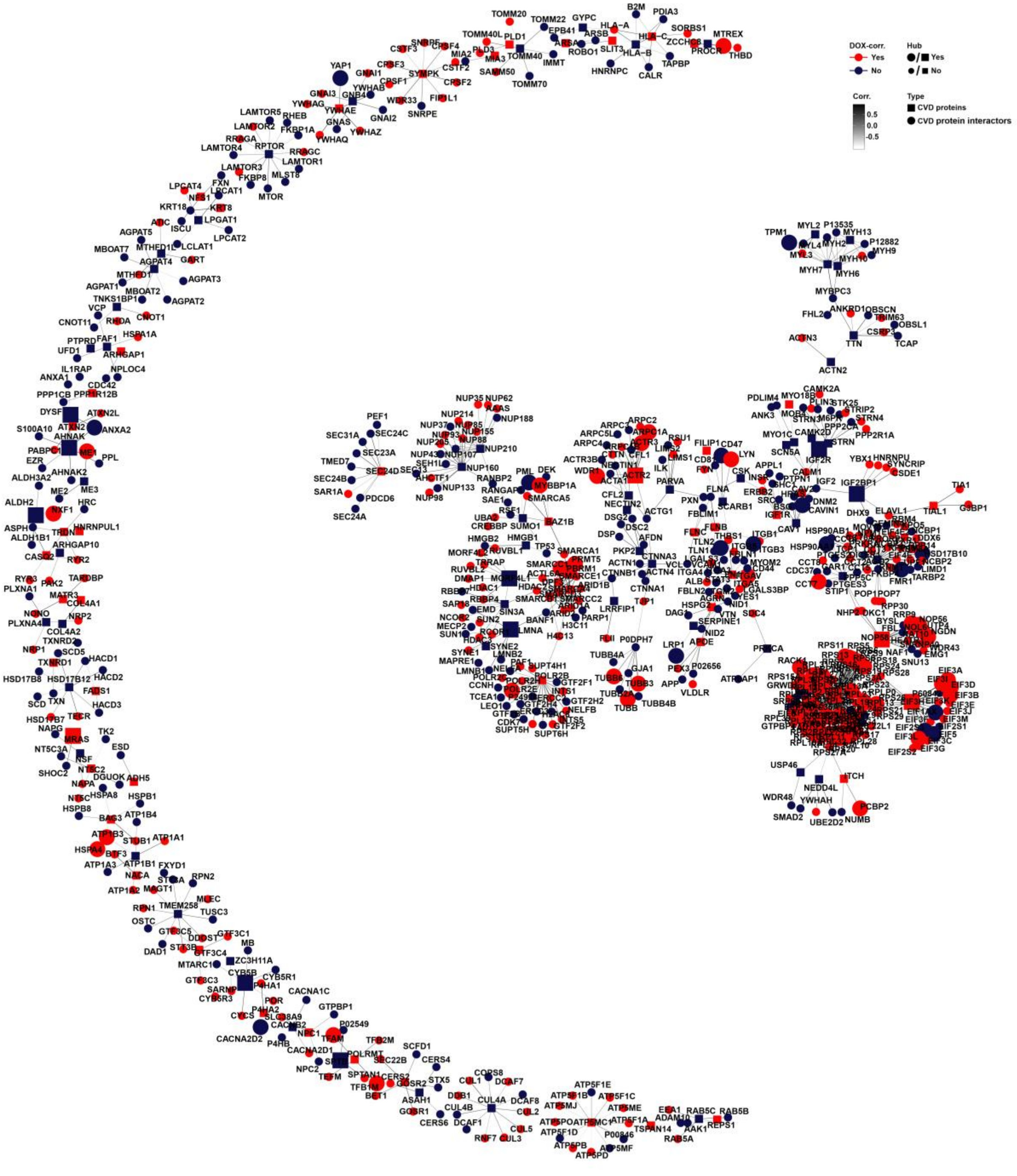

Figure S16: Functionally annotated CVD-PPI network within the context of the DNA damage response, related to Figure 6. Protein-protein interaction (PPI) network for CVD risk proteins (square) and CVD risk protein interactors (circle) expressed within the co-expression network. Edges represent the weighted correlation between interaction pairs. Node size indicates if a protein is a hub (large icon) or not a hub (small icon) protein. Color denotes if a protein is DOX-correlated (red) or not DOX-correlated (blue).

Supplemental references

[S1] Uhlén, M., Fagerberg, L., Hallström, B.M., Lindskog, C., Oksvold, P., Mardinoglu, A., Sivertsson, Å., Kampf, C., Sjöstedt, E., and Asplund, A. (2015). Tissue-based map of the human proteome. Science 347, 1260419.

[S2] Jiang, L., Wang, M., Lin, S., Jian, R., Li, X., Chan, J., Dong, G., Fang, H., Robinson, A.E., and Aguet, F. (2020). A quantitative proteome map of the human body. Cell 183, 269-283. e219.

[S3] Szklarczyk, D., Kirsch, R., Koutrouli, M., Nastou, K., Mehryary, F., Hachilif, R., Gable, A.L., Fang, T., Doncheva, N.T., and Pyysalo, S. (2023). The STRING database in 2023: protein–protein association networks and functional enrichment analyses for any sequenced genome of interest. Nucleic acids research 51, D638-D646.
